# The vaginal microbiota of women living with HIV on suppressive antiretroviral therapy and its relation to high-risk human papillomavirus infection

**DOI:** 10.1186/s12866-023-02769-1

**Published:** 2023-01-19

**Authors:** Monserrat Chávez-Torres, Maria Gómez-Palacio-Schjetnan, Gustavo Reyes-Terán, Olivia Briceño, Santiago Ávila-Ríos, Karla Alejandra Romero-Mora, Sandra Pinto-Cardoso

**Affiliations:** 1 Departamento de Investigación en Enfermedades Infecciosas, Instituto Nacional de Enfermedades Respiratorias Ismael Cosío Villegas, Calzada de Tlalpan 4502, Colonia Sección XVI, Tlalpan, 14080 Ciudad de México, México; 2 Comisión Coordinadora de Institutos Nacionales de Salud Y Hospitales de Alta Especialidad, Secretaría de Salud, Ciudad de México, México

**Keywords:** Human immunodeficiency virus, Antiretroviral therapy, Human papillomavirus, High-risk HPV, Cervical cytology, Genital inflammation, Vaginal microbiota, Vaginal pH, Immune activation, *Lactobacillus iners*

## Abstract

**Background:**

Few studies have investigated the vaginal microbiota (VM) in women living with HIV (WLWH) in the context of high-risk human papillomavirus (HR-HPV) infection, even though WLWH are at an increased risk of HPV-related malignancies, including cervical cancer. To explore the impact of HIV and HPV infection on the VM in WLWH, we determined the prevalence of HR-HPV infection and cervical cytologic abnormalities in a cohort of 44 WLWH and 39 seronegative-women (SNW), characterized the vaginal microbiota by 16S sequencing, assessed genital inflammation and systemic immune activation by multiplex bead assay and flow cytometry, respectively. Finally, we explored relationships between bacterial richness and diversity, the top 20 bacterial genera, genital inflammation and systemic immune activation.

**Results:**

We found that HR-HPV prevalence was similar between WLWH and SNW. High-grade squamous intraepithelial lesions (HSIL) were only detected in WLWH negative for HR-HPV infection. In regression analyses, no risk factors were identified. Women co-infected with HIV and HR-HPV had the highest level of systemic immune activation, and these levels were significantly different compared with SNW without HR-HPV infection. *Lactobacillus iners* was the dominant *Lactobacillus* species in WLWH and SNW alike.

**Conclusion:**

We found no evidence of differences in vaginal microbial richness and diversity, microbial community structure, and genital inflammation by HIV, HPV, or HIV and HPV status.

**Supplementary Information:**

The online version contains supplementary material available at 10.1186/s12866-023-02769-1.

## Background

The vaginal microbiota (VM) has been extensively characterized in gynecological and obstetrics complications, including genital tract infections like bacterial vaginosis (BV), malignancies (cervical cancer), acquisition and transmission of sexually transmitted diseases (STDs), and other physiological conditions (pregnancy, menopause) [1, 2]. Low diversity and *Lactobacillus* dominance are the hallmark of an optimal vaginal microbiota [3]. *Lactobacillus* species (spp.) produce lactic acid, a potent microbicide that plays an important role in maintaining the acidic pH of the vagina; this microenvironment is responsible for promoting homeostasis by inactivating pathogens like human immunodeficiency virus (HIV) and herpes simplex virus (HSV) type 2 and excluding anaerobes [4]. In contrast, a non-optimal (also termed dysbiotic) vaginal microbiota is highly diverse, polymicrobial in nature, and dominated by anaerobes, such as *Gardnerella vaginalis*, *Atopobium vaginae*, *Prevotella bivia*, among others [5]. This dysbiotic microbiota, commonly known as community state type (CST) IVa (*G. vaginalis* dominance) and IVb (highly diverse, no particular species dominance [6]), is strongly associated with genital inflammation [7] and epithelial damage [8]. Many host and external factors influence the composition of the VM, including oral contraception (estrogens [9], sexual activity and practices, smoking, menses, pregnancy, menopause, and to a lesser extent antibiotic use [5]).

The role of the VM in HIV risk acquisition has been demonstrated [10, 11]. Studies have shown that dysbiotic vaginal microbial communities, also referred to as bacterial vaginosis-associated bacteria (BVAB), increase the risk of HIV acquisition and modify the efficacy of antiretroviral-based microbicides [12, 13]. In sharp contrast, studies in women living with HIV (WLWH) are limited [14, 15]. Studies have shown that WLWH have non-optimal vaginal microbiotas, that are associated with vaginal inflammation, HIV shedding and increased risk of sexual transmission to their male partners (WLWH who do not achieve virologic suppression [16–18]). The impact of antiretroviral therapy (ART) on the vaginal microbiota is currently unknown [19]. Fewer studies have investigated the VM of WLWH in the context of human papillomavirus (HPV) infection [14, 20, 21], although evidence suggests that HIV enhances HPV pathogenicity [22] and WLWH are at an increased risk of HPV-related malignancies, including cervical cancer [23], even those on ART [24, 25]. Furthermore, the impact of ART on HPV-related cervical pathology remains to be elucidated [22, 26]. Understanding the complex interplay between HIV, HR-HPV, the VM and ART is relevant and necessary to inform policy makers on adequate screening programs and clinical management of WLWH to alleviate the burden of cervical cancer in this vulnerable at-risk population [27].

The aim of this study was to characterize the VM of WLWH with complete virologic suppression, to explore relationships between vaginal inflammation, systemic immune activation, demographic and clinical data, and compare them to SNW, in the context of high-risk HPV infection (HR-HPV). To our knowledge, this is one of the first studies to include both WLWH and SNW with or without HR-HPV infection.

## Results

### Characteristics of study participants

A total of 83 women were included, 44 were WLWH and 39 were SNW. Characteristics are summarized in Table 1 and Additional Files 1 and 2. The median age was 42 (21–69) years; WLWH were older than SNW (median of 44 versus 39 years, *p* = 0.029). Other than age, no differences were found with regards to age at sexual debut, previous history of STDs, type of STDs, vaginal pH, smoking, or alcohol use. SNW had a higher total number of sexual partners compared to WLWH (*p* = 0.002), and were less likely to use a condom (*p* = 0.001). Data relating to reproductive health and sexual practices are summarized in Additional File 1. More than half of women, SNW and WLWH alike, presented with gynecological symptoms (59.04%) and these included intermenstrual bleeding (16.87%), vaginal discharge (44.58%), dyspareunia (24.1%), pelvic pain (30.12%) and postcoital bleeding (9.64%). There were no differences by HIV status. No women in our study reported using oral hormonal contraception.

**Table 1:** Cohort description

	**All**	**SNW**	**WLWH**	***P* value**
**Number**	83	39	44	NA
**Age (years)**				
Median (min, max)	42 (21–69)	40 (22–69)	44 (21–65)	0.029*
**Age at sexual debut (years)**				
Median (min, max)	17 (6–36)	17 (13–36)	18 (6–25)	0.681
**Total number of sexual partners**				
Median (min, max)	3 (1–20]	4 (1–20)	2 (1–15)	0.002*
**Nb of sexual partners in the last year**				
Mean (min, max)	1 (0–4)	1.3 (0–4)	0.9 (0–4)	0.013*
**Condom use,** n (%)				
Always	12 (14.46)	5 (12.82)	7 (15.91)	0.001*
Sometimes	45 (54.22)	29 (74.36)	16 (36.36)	
Never	26 (31.33)	5 (12.82)	21 (47.73)	
**Vaginal pH**				
Median (min, max)	5.5 (5–9)	5.5 (5–8)	5.5 (5–9)	0.431
**Previous STDs,** n (%)				
No	64 (77.11)	31 (79.49)	33 (75)	0.794
Yes	19 (22.89)	8 (20.51)	11 (25)	
**STD,** n (%)				
Candidiasis	31 (37.4)	18 (46.15)	13 (29.54)	0.320
Chlamydia	3 (3.6)	3 (7.69)	0 (0)	
Genital herpes	4 (4.8)	1 (2.56)	3 (6.8)	
Genital warts	7 (8.4)	3 (7.69)	4 (9.09)	
Syphilis	1 (1.2)	0 (0)	1 (2.27)	
Others	8 (9.6)	5 (12.86)	3 (6.81)	
**Menopause,** n (%)				
No	68 (82.93	34 (94.44)	34 (79.07)	> 0.999
Yes	14 (17.07)	5 (5.56)	9 (20.93)	
Missing data	1	0	1	
**Smoking,** n (%)				
Current smoker	10 (12.05)	6 (15.38)	4 (9.09)	0.663
Never smoked	54 (65.06)	24 (61.54)	30 (68.18)	
Former smoker	19 (22.89)	9 (23.08)	10 (22.73)	
**Alcohol use,** n (%)				
Light	73 (90.12)	34 (87.18)	39 (92.86)	0.472
Moderate	8 (9.88)	5 (12.82)	3 (7.14)	
Missing data	2	0	2	
**Number of 16S sequences**				
Median	175,459	172,039	176,431	0.2748
[IQR]	[114022–247647]	[101007–221992]	[118423–303646]	

Data are expressed as n (%), unless stated otherwise. Wilcoxon Rank Sum test was used to compare continuous variables and chi square or Fisher's exact test for categorical variables. * *p* < 0.05 (statistical significance)

*Abbreviations*: *IQR* Interquartile range, *max* Maximum, *min* Minimum, *NA* Not applicable, *Nb* Number, *SNW* Seronegative women, *STDs* Sexually Transmitted Diseases, *WLWH* Women living with HIV

### Immune status and ART exposure in WLWH

Data on HIV clinical parameters are summarized in Additional file 2. The median CD4 + T-cell count was 529.5 [307.3–728.8] cells/mm^3^. Twenty-three WLWH had CD4 > 500 cells/mm^3^ (52.27%), 17 (38.64%) had 200 < CD4 < 500 and only 4 (9.09%) had CD4 below 200. All WLWH had undetectable viremia (< 40 copies/mL); 40 were on antiretroviral treatment (ART) and 4 were elite controllers (defined as WLWH with undetectable HIV-1 viral load in the absence of ART). The most frequent ART regimen was based on non-nucleoside reverse transcriptase inhibitors (NNRTIs, *n* = 24, 54.55%) followed by protease inhibitors (PIs, *n* = 12, 27.27%). WLHW were diagnosed in late stages of HIV infection as reflected by their CD4 nadir (median 105.5 [29.25–240] cells/mm^3^). All women tested negative for hepatitis B and C viruses.

### Prevalence of high-risk HPV infection

As summarized in Table 2, 26 (31.33%) were positive for HR-HPV, with two women being positive for HPV16 infection (1 SNW: single infection and 1 WLHW: co-infection). HPV18 was not detected in our cohort. Cervical cytology results reported that 5 (6.02%) women had low-grade squamous intraepithelial lesions (LSIL) and 2 (2.41%) had high-grade squamous intraepithelial lesions (HSIL). HSIL were only found in WLWH, while LSIL were found in 4 SNW and 1 WLWH. WLWH with HSIL or LSIL tested negative for HR-HPV, while two SNW with LSIL tested positive for HPV. There were no differences with regards to HR-HPV infection and cervical cytology between WLWH and SNW. We searched for differences in well-known risk factors associated with HPV infection, including age, sexual history, smoking, HIV, previous STD and parity between HPV-negative (HPVN) and HPV-positive (HPVP) women (Additional file 3) and found none. Additionally, no risk factors were identified when performing logistic regression analyses.

**Table 2:** High-Risk HPV infection and cervical cytology

	**All**	**SNW**	**WLWH**	***P* value**
**Number**	83	39	44	
**HPV infection**				
Positive	26 (31.33)	13 (33.33)	13 (29.55)	0.813
Negative	57 (68.67)	26 (66.67)	31 (70.45)	
**HR-HPV genotypes**^**a**^				
HPV16	1 (3.85)	1 (7.69)	0 (0)	0.367
HPV16 and others HR-HPV	1 (3.85)	0 (0)	1 (7.69)	
Others hrHPV	24 (92.31)	12 (92.31)	12 (92.31)	
**Cervical cytology**^**b**^				
NILM	66 (90.41)	30 (88.24)	36 (92.31)	0.697
Abnormal cervical cytology	7 (9.59)	4 (11.76)	3 (7.69)	
Type of abnormal cytology^**b**^				
LSIL	5 (6.85)	4 (11.76)	1 (2.56)	
HSIL	2 (2.74)	0 (0)	2 (5.13)	
**HPV vaccine**^**c**^				
NO	43 (89.58)	29 (93.55)	14 (82.35)	0.330
YES	5 (10.42)	2 (6.45)	3 (17.65)	

Data are expressed as n (%). Fisher's exact test was used to compare categorical variables

*Abbreviations*: *HPV* Human Papillomavirus, *HSIL* High-grade squamous intraepithelial lesions, *HR-HPV* High Risk Human Papillomavirus, *LSIL* Low-grade squamous intraepithelial lesions, *NILM* Negative for intraepithelial lesion or malignancy, *SNW* Seronegative women, *WLWH* Women living with HIV

^a^HPV detection assay can detect 14 high risk HPV genotypes: 16, 18, 31, 33, 35, 39, 45, 51, 52, 56, 58, 59, 66, 68 and partially genotype 16, 18 from other 12 high risk genotypes. Others HR-HPV refers to the 12 HR-HPV detected by the assay

^**b**^Cytology screening was reported according to the Bethesda system. Cervical cytology results were only available for 73 women (34 SNW and 39 WLWH). Frequencies of LSIL and HSIL were calculated overall (All, *n* = 83, SNW, *n* = 39 and WLWH, *n* = 44 and not within women with abnormal cytology)

^**c**^Data on HPV vaccination were missing in 35 women (8 SNW and 27 WLWL)

### Genital inflammation and systemic immune activation

**Fig. 1:**
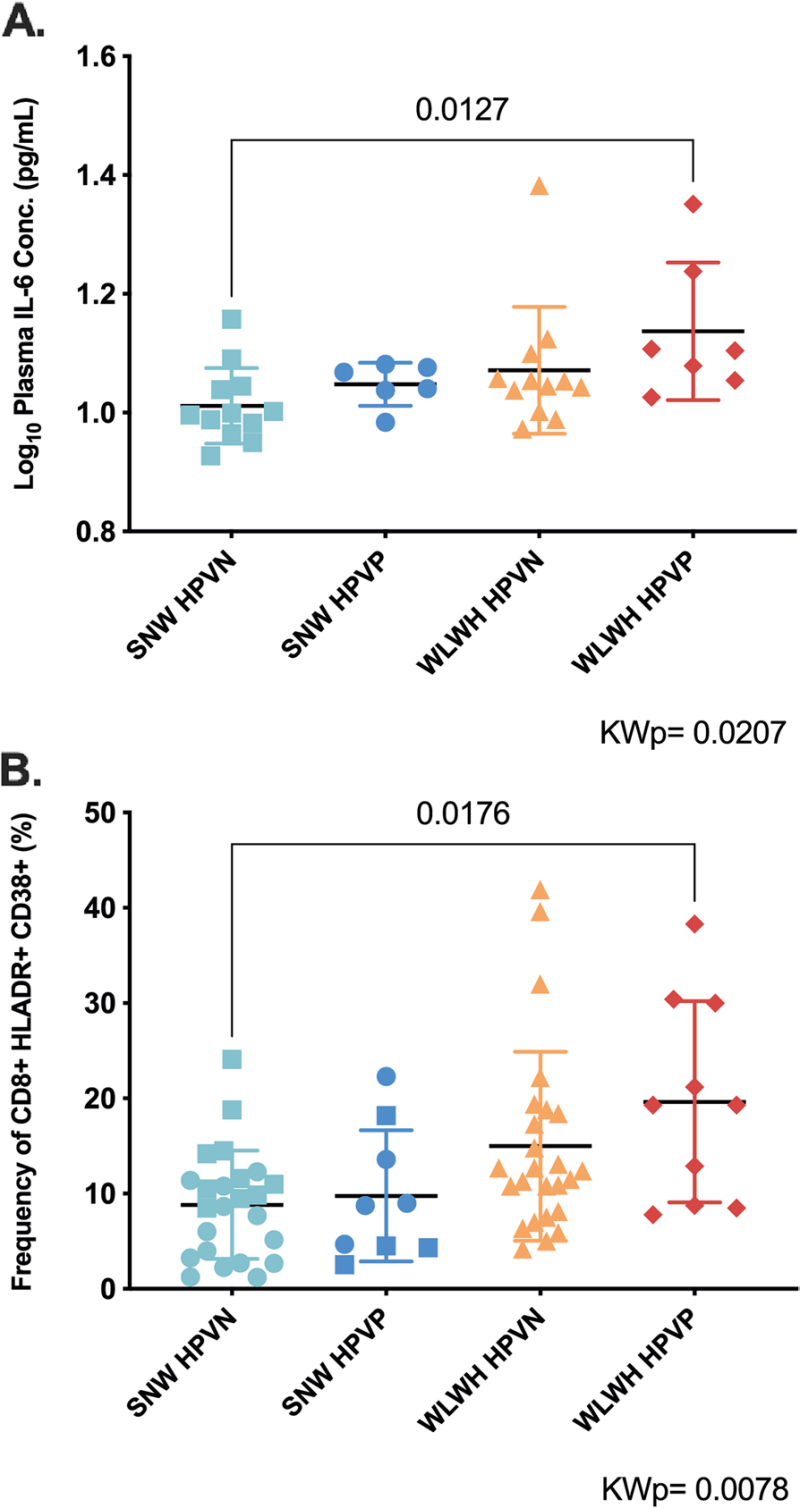
Plasma IL-6 and CD8 + T cell activation are highest in women living with HIV with HR-HPV infection. Legend: Scatter plot showing the median and interquartile range of A. The log_10_ plasma IL-6 concentrations and B. The frequency of CD8 + HLADR + CD38 + (%) in seronegative women (SNW) and women living with HIV (WLWH) with or without HPV infection. Kruskal–Wallis test was used to compare groups and *p* values were adjusted for multiple comparisons using Dunn's multiple comparisons test. *p < 0.05 (statistical significance). Only significant adjusted *p* values are shown. Statistical analysis and graphs were done in GraphPad Prism 9. Abbreviations: CD: cluster of differentiation, HLA: human leukocyte antigen, HIV: human immunodeficiency virus, HPV: human papillomavirus, HPVP: HPV positive, HPVN: HPV negative, KWp = Kruskal–Wallis p, ml = milliliter, pg = picogram, SNW: seronegative women, WLWH: women living with HIV, %: frequency

First, we determined the level of inflammation locally (cytobrush supernatant) and systematically (plasma) by quantifying soluble markers (a panel of 30 cytokines, chemokines, and growth factors). We found no differences in genital inflammation when stratifying by HIV status or HPV status (Additional file 4). Plasma levels of IL-6, IL-8, IP-10, MCP-1, MIG, and Eotaxin were elevated in WLWH compared to SNW (Additional file 5). When stratifying by HPV infection, only IL-6 was elevated in women with HR-HPV infection. Interestingly, WLWH with HR-HPV infection (WLWH HPVP) had the highest plasma levels of IL-6; this latter being significantly different compared with SNW negative for HPV infection (*p* = 0.0127, Fig. 1A and Additional file 6). Next, we also analyzed the co-expression of HLADR and CD38, two classical markers of T cell activation, on both peripheral blood CD4 + T and CD8 + T cells by flow cytometry (Additional file 7). The frequency of CD8 + T cell activation was higher in WLWH (*p* = 0.0012), while the frequency of CD4 + T cell activation was lower (*p* < 0.0001) compared with SNW. No differences in systemic immune activation were observed when stratifying by HPV infection. WLWH with HR-HPV infection had the highest levels of CD8 + T cell activation and these levels were significantly different compared with SNW negative for HPV infection (*p* = 0.017, Fig. 1B and Additional file 8).

### The vaginal microbiota in HIV and HR-HPV infection

**Fig. 2:**
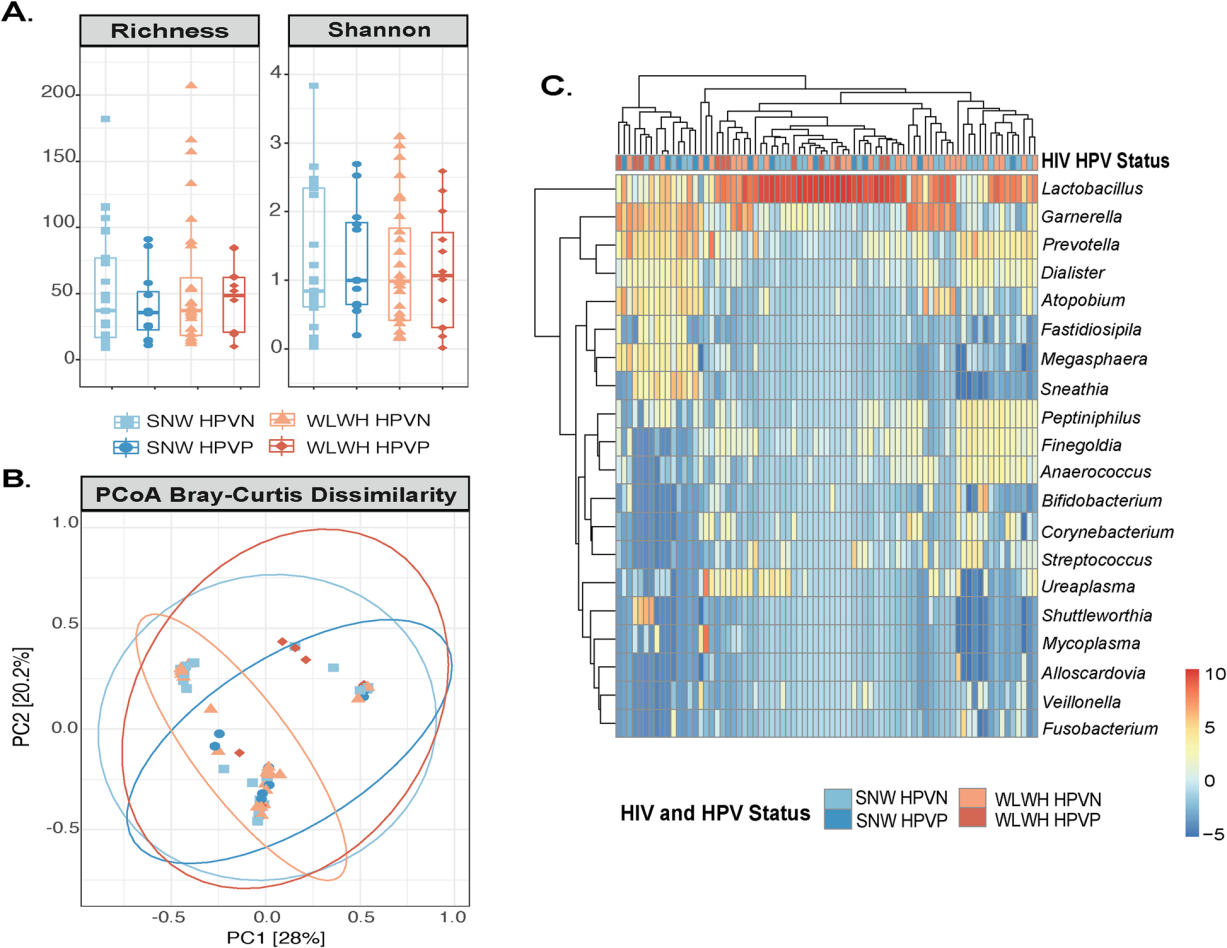
The vaginal microbiota of women living with HIV and seronegative women with or without HR-HPV infection. Legend: **A**. Boxplots showing the median and interquartile range of two alpha diversity metrics: richness (number of observed species) and shannon (richness and evenness). Groups were compared using Kruskal–Wallis test and *p* values adjusted for multiple comparisons (KWp = 0.933 for richness, and KWp = 0.936 for shannon). No difference in alpha diversity was observed when stratifying by HIV status, HPV status or both. **B** .Clustering of microbial communities was visualized using principal coordinate analysis (PCoA, Bray–Curtis dissimilarity) and differences were assessed by PERMANOVA after testing for homogeneity of dispersions (betadisper). Pairwise comparisons revealed that microbial communities of WLWH with HPV infection (WLWH HPVP) were marginally different from those of WLWH without HPV infection (WLWH HPVN, R^2^ = 0.049, *p* = 0.047), with no other difference being observed (SNW HPVN vs SNW HPVP, *R*^*2*^ = 0.024, *p* = 0.472; WLWH HPVN vs SNW HPVN *R*^*2*^ = 0.026, *p* = 0.162; WLWH HPVP vs SNW HPVP, *R*^*2*^ = 0.029, *p* = 0.678). **C**. Heatmap showing the top 20 genera stratified by HIV and HPV status. The heatmap was generated using ampvis2 in R. Relative abundances were centered log-ratio transformed. Abbreviations: HIV: human immunodeficiency virus, HPV: human papillomavirus, HPVN: HPV negative, HPVP: HPV negative, PCoA: principal coordinate analysis, PERMANOVA: Permutational multivariate analysis of variance, *R*^*2*^ = R squared, SNW: seronegative women, WLWH: women living with HIV

We characterized the vaginal microbiota using 16S rDNA sequencing. Vagina swabs from 6 women were not available, leaving 37 SNW and 40 WLWH for subsequent analyses. Two metrics were calculated to estimate alpha diversity: shannon (richness and evenness) and richness (number of observed species). No difference in alpha diversity (Fig. 2A and Additional file 9) was observed when stratifying by HIV, HPV or both HIV and HPV status. Clustering of microbial communities was visualized using principal coordinate analysis (PCoA, Bray–Curtis dissimilarity) and differences were assessed by PERMANOVA after testing for homogeneity of dispersions (Fig. 2B and Additional file 10). We found no significant differences when considering HIV status (*R*^*2*^ = 0.010, *p* = 0.626), HPV status (*R*^*2*^ = 0.021, *p* = 0.104) or both (*R*^*2*^ = 0.047, *p* = 0.235). Pairwise comparisons revealed that microbial communities of WLWH with HPV infection (WLWH HPVP) were marginally different from those of WLWH without HPV infection (WLWH HPVN, *R*^*2*^ = 0.049, *p* = 0.047), with no other differences being observed (SNW HPVN vs SNW HPVP, *R*^*2*^ = 0.024, *p* = 0.472; WLWH HPVN vs SNW HPVN *R*^*2*^ = 0.026, *p* = 0.162; WLWH HPVP vs SNW HPVP, *R*^*2*^ = 0.029, *p* = 0.678). The relative abundance of the 20 most abundant genera is shown in Fig. 2C and Additional file 11. Overall, the most abundant genera included *Lactobacillus* (60.83%), *Gardnerella* (14.49%), *Prevotella* (6.92%), *Atopobium* (2.58%), *Sneathia* (1.97%), *Bifidobacterium* (1.76%), *Shuttleworthia* (1.64%), *Mycoplasma* (1.05%), representing 97.62% of all genera. Interestingly, both women with HPV16 (1 SNW and 1 WLHW) had polymicrobial communities, dominated by *Gardnerella* and *Atopobium* (for WLHW), and *Gardnerella*, *Atopobium, Prevotella* and *Sneathia* (for SNW), and depleted in *Lactobacillus* (Additional file 11). At species level, we identified *Lactobacillus iners*, *L. jensenii*, *L. gasseri*, *L. reuteri*, however we did not find *L. crispatus*. We could also identify *G. vaginalis*, *P. bivia*, *A. vaginae*, *S. amnii*, *B. breve*, among others, as summarized in Table 3 and Additional file 12.

**Table 3:** Mean relative abundance (%) of the top 20 genera and the most abundant species within each genus in women living with HIV and seronegative women with or without HR-HPV infection

	**SNW HPVN**	**SNW HPVP**	**WLWH HPVN**	**WLWH HPVP**
Actinobacteria				
*Atopobium*	1.4	5.2	2.6	2.2
*A. vaginae*	1.3	5.2	2.6	2.1
*Gardnerella*	12.9	24.4	17	18
*G. vaginae*	12.9	24.4	17	18
*Alloscardia*	0	0	1.9	0
*A. omnincolens*	0	0	1.9	0
*Corynebacterium*	1.7	0.2	0.2	0.2
*C. coyleae*	0.9	0	0	0
Firmicutes				
*Lactobacillus*	55.3	38	54.7	63.6
*L. iners*	35.9	14.4	34.2	52.3
*L. jensenii*	8.5	8.8	6.4	7
*L. gasseri*	2	4.6	11.2	0.1
*L. reuteri*	7.4	0.5	1.7	3.7
*L. delbrueckii*	0	8.3	0	0
*L. coleohominis*	1.5	1	0	0.2
*L. johnsonii*	0	0.3	1.2	0
*Bifidobacterium*	2.1	0	3.5	0
*B. breve*	2.1	0	2.7	0
*Shuttleworthia*	2	0	0	6.6
*Mycoplasma*	0.1	6.7	0.5	0
*M. hominis*	0.1	6.7	0	0
*Ureaplasma*	0.1	7.2	0.5	0.1
*U. parvum*	0.1	7.2	0.1	0.1
*U. urealyticum*	0	0	0.4	0
*Megasphaera*	0.7	1	0.5	1.1
*Dialister*	2	1	0.6	0.4
*D. propionicifaciens*	0.6	0.1	0.5	0.2
*Peptoniphilus*	0.8	0.1	0.7	0
*Finegoldia*	1	0.1	0.5	0.1
*Streptococcus*	1.7	0.1	0.4	0
*S. anginosus*	1.4	0.1	0.4	0
*Anaerococcus*	1.3	0.1	0.7	0
*A. mediterraneesis*	0.8	0.1	0.1	0
*Veillonella*	0.1	0	1	0
*V. montpellierensis*	0	0	0.7	0
*Fastidiosipila*	0.6	0.3	0.1	0.2
Bacteroidetes				
*Prevotella*	10.6	8.7	9.6	3.1
*P. bivia*	5.5	1.1	5.5	0.8
*P. timonensis*	1.8	0.7	2.3	0.7
*P. amnii*	1.5	2.4	0	0.1
*P. buccalis*	0.5	3.7	0.5	0.8
Fusobacteria				
*Sneathia*	3.9	3.3	0.1	1.1
*S. amnii*	3.6	3.2	0.1	0.9
*Fusobacterium*	0.1	0	0.4	0
*F. nucleatum*	0	0	0.4	0

*Abbreviations*: *HIV* Human immunodeficiency virus, *HR-HPV* High-risk Human Papillomavirus, *SNW* Seronegative women, *WLWH* Women living with HIV

**Fig. 3:**
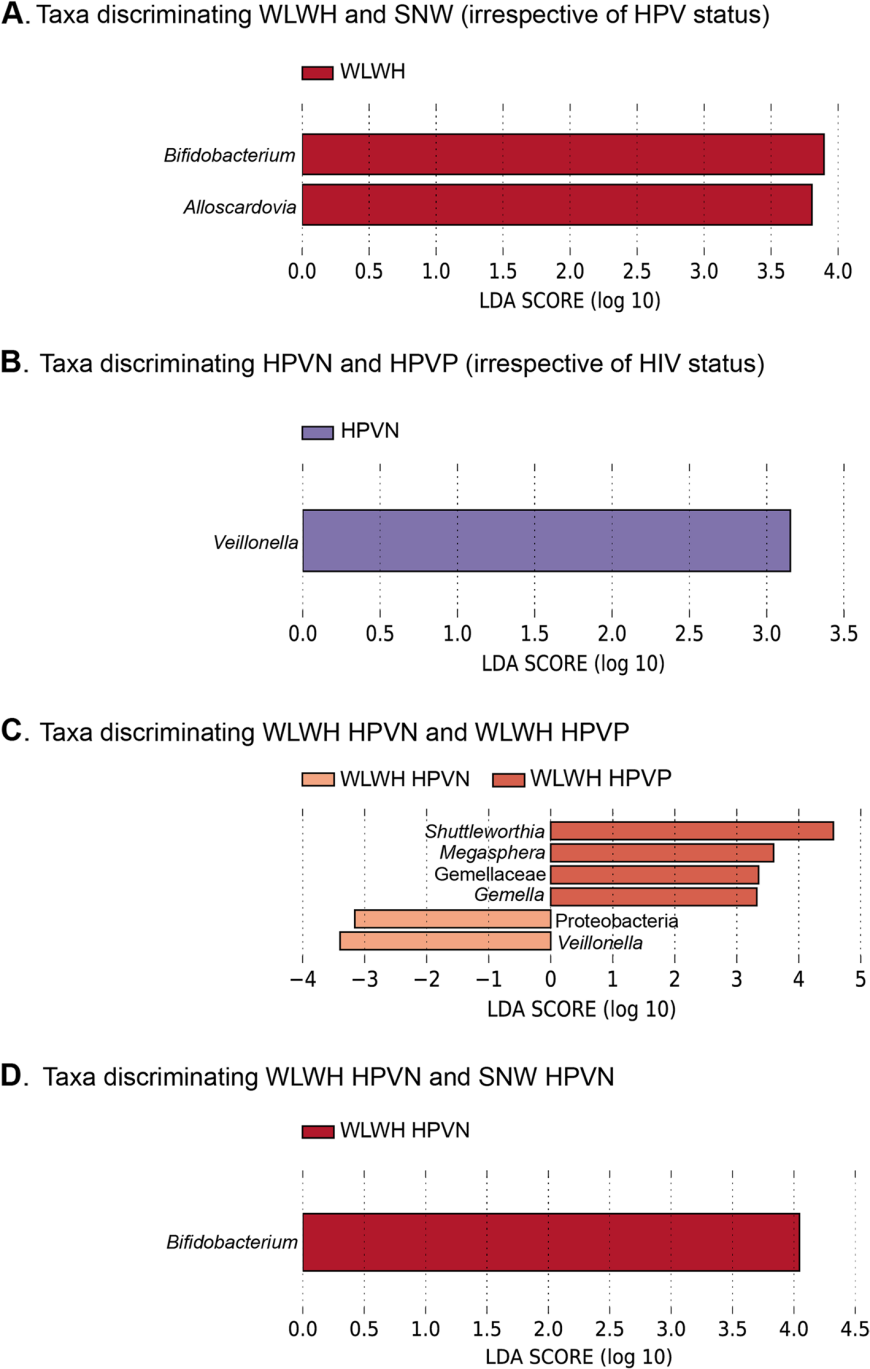
Differentially expressed taxa in women living with HIV and seronegative women with or without HPV infection as determined by Linear Discriminant Analysis Effect Size (LEfSe). Legend: Linear discriminant analysis plots showing differentially discriminant taxa identified in A. WLWH and SNW irrespective of HPV infection; B. HPVN and HPVP women irrespective of HIV status; C. WLWH only; and D. HPVN women only. LEfSe analyses were performed at https://huttenhower.sph.harvard.edu/galaxy/, with the following parameters: LDA threshold 3 or higher, alpha value for factorial Kruskal–Wallis test 0.05 and alpha value for pairwise Wilcoxon test 0.05. The ASV table was filtered prior to LEfSe analysis, removing ASVs not seen more than 5 times in at least 5% of samples, to avoid including ASVs with small means and outliers. Abbreviations: ASVs: amplicon sequences variants, LDA: Linear Discriminant Analysis, HIV: human immunodeficiency virus, HPV: human papillomavirus, HPVP: HPV positive, HPVN: HPV negative, SNW: seronegative women, WLWH: women living with HIV

Next, we used LEfSe to identify discriminative taxa (linear discriminant analysis threshold of 3.0 or higher, Fig. 3). When considering all 4 groups (SNW HPVN, SNW HPVP, WLWH HPVN and WLWH HPVP), no discriminative taxa were identified. When considering WLWH and SWN irrespective of their HPV status (Fig. 3A), two genera were found to be differentially expressed in WLWH: *Bifidobacterium* (LDA = 3.93, *p* = 0.011) and *Alloscardovia* ((LDA = 3.90, *p* = 0.049). When considering HPVN and HPVP irrespective of their HIV status, *Veillonella* was identified in HPVN ((LDA 3.19, *p* = 0.043, Fig. 3B). When only considering WLWH, three genera were identified in WLWH with HPV infection: *Gemella* (LDA = 3.32, *p* = 0.006), *Megasphaera* (LDA = 3.59, *p* = 0.013), and *Shuttleworthia* (LDA = 4.55, *p* = 0.03), while *Veillonella* (LDA -3.39, *p* = 0.042) was identified in WLWH without HPV infection (Fig. 3C). Lastly, *Bifidobacterium* was found to discriminate WLWH without HPV infection from SNW without HPV infection (LDA = 4.044, *p* = 0.006, Fig. 3D). No discriminant features were identified in SNW only and HPVP women only.

### Relationships between bacterial taxa and soluble and cellular markers of inflammation and immune activation

Finally, we explored relationships between the VM and soluble and cellular markers of inflammation and immune activation, as well as demographic and clinical data. We only considered correlations with a Spearman rho >±0.6 and *p* < 0.01. Overall, we found inverse correlations between *Lactobacillus* and *Gardnerella*, *Prevotella*, *Dialister*, and *Atopobium*, as expected, and positive correlations between facultative anaerobes as shown in Additional file 13. No correlations were found between the VM and soluble markers or cellular markers of inflammation, vaginal pH or any other clinical data.

## Discussion

Data regarding the VM in women co-infected with HIV and HPV is limited. In our study, we aimed to understand the impact of both HIV and HPV infection on the VM and related these to genital inflammation and systemic immune activation.

Our results are in accordance with previous studies that found no differences in the VM of HIV-positive and HIV-seronegative women [14, 20, 21, 28]. To be precise, we found no difference in either vaginal microbial diversity (alpha diversity) or microbial community structure (beta-diversity). Interestingly, 2 genera were found to discriminate WLWH from SNW (using LEfSe): *Bifidobacterium* and *Alloscardia*. *Alloscardia omnincolens* is a pathogen that together with *Prevotella bivia* and *Streptococcus anginous* are thought to be responsible for aerobic vaginitis, a vaginal dysbiotic state that is associated with clinically overt inflammation [29]. *Bifidobacterium* was found in the VM of 3 women, 1 SNW and 2 WLWH, and these 3 women were HPV-negative. The VM of these 3 women was polymicrobial in nature and *Lactobacillus*-depleted. Interestingly, because *Bifidobacterium* is capable of colonizing the vagina and produce lactic acid, a protective role similar to the one played by *Lactobacillus* has been suggested [30, 31]. Bifidobacterial species are considered probiotics. In our study, no woman with HPV infection was colonized with *Bifidobacterium*, which may indicate that *Bifidobacterium* could have a protective role against HPV infection, although we are aware that the number of women with *Bifidobacterium* in our study was very low, making it difficult to draw conclusions. We also found that *Bifidobacterium* discriminated WLWH without HPV infection from SNW without HPV infection. Interestingly, *Bifidobacterium* correlated inversely with *Lactobacillus*, and positively with *Alloscardovia*, Anaerococcus, *Prevotella* and *Dialister*. Whether the vaginal microbiota of WLWH offers a preferential microenvironment for the growth of *Bifidobacterium* or the presence of *Bifidobacterium* is a compensatory mechanism in the absence of *Lactobacillus* remains to be elucidated. It is worth mentioning that in vitro studies have shown that *Bifidobacterium* isolated from a vaginal swab of a healthy woman exerted strong antimicrobial activity against urogenital and enteric pathogens [32]. Further studies are needed to clarify the clinical significance of *Bifidobacterium* in the vaginal microbiota. Also, very little is known about *A. omnincolens*. Studies have started to clarify the role of *A. omnincolens*, and have alluded to the fact that *A. omnincolens* does not appear to contribute to urgency urinary incontinence [33, 34].

In our cohort, *L. iners* was the predominant *Lactobacillus* species, however, its dominance was not associated with either HIV or HPV infection or both, even though WLWH with HPV infection had the highest mean relative abundance of *L. iners*. The role of *L. iners* in maintaining an optimal VM is debatable [35] as it has been shown that *L. iners* is a transitional species that colonizes the vagina after the vaginal microenvironment is disturbed. *L. iners* is considered less protective than *L. crispatus* [36], and is associated with a higher prevalence of STDs [17, 37]. At the same time, *L. iners* is found in the VM in women of different ethnic groups (other than Caucasian) [6, 38]. Our cohort was composed of Mexican Mestizo women. The fact that we found predominance of *L. iners* and high vaginal pH (5 and above) is in accordance with previous observations [6]. Also, *L. iners* has been found in Hispanic and non-Hispanic women with HPV [39]. The predominance of *L. iners* and absence of *L. crispatus* could have relevant clinical implications for Mexican WLWH and SNW alike, since in Mexico, cervical cancer is the most common cancer among women [40]. Furthermore, *L. iners* has been associated with increased risk of STDs [17, 37] and instability of the VM [41].

The prevalence of HR-HPV in WLWH was comparable to SNW. Few studies have reported HPV prevalence in women in Mexico, and even less in WLWH [42]. The overall prevalence of HR-HPV in our study was 31.33%, which is higher than the one reported by Torres et al. [40] and López Rivera et al. [42]. Torres and colleagues surveyed female users affiliated to the Institute of Security and Social Services for State Workers (ISSSTE) and found an overall prevalence of 13% [40]. López and colleagues reported an HPV prevalence of 9.1% [42]. Discrepancies between our results and these two studies could be due to different HPV detection assays used in each study. Also, HPV prevalence varies within population sub-groups, in particular if differences in HPV-associated risk-factors are observed (age, smoking, use of hormonal contraception, among others). Interestingly, traditional risk factors associated with HR-HPV infection like smoking were not observed in our cohort. In this study, 9.59% of women had abnormal cervical cytology (5 LSIL and 2 HSIL). Abnormal cervical cytologic were found in only 2 HPV-positive women (2 SNW with LSIL). Given the higher HR-HPV prevalence found in our study, these women could be at an increased risk of cervical cancer, and were encouraged to follow the national program for early detection of cervical cancer (prevention through early detection with the Papanicolaou test, in conjunction with appropriate treatment of detected lesions) [43]. As mentioned previously, WLWH in this study had abnormal cervical cytology, predominantly LSIL. Almost half of WLWH (*n* = 21) had not normalized their CD4 T cell count even on suppressive ART (CD4 < 500 cells). Also, WLWH were chronically-infected with HIV prior to start ART (their median nadir CD4 was 105 cells/mm^3^, additional file 2)). Given that CD4 T cell count is a risk-factor for HPV infection, and the recurrence and progression rate of cervical neoplasia is high in immunocompromised WLWH, increased vigilance and screening are warranted [27]. Also, immunocompromised WLWH might benefit from a more aggressive clinical management compared to their immunocompetent counterpart [44]. The impact of ART on HPV-related cervical pathology remains uncertain [22, 26]. Continued efforts to understand the complex interplay between HIV, HR-HPV, the VM and ART are warranted to generate relevant and necessary information to alleviate the burden of cervical cancer in this vulnerable at-risk population [27].

Results from LEfSe analysis indicated that *Shuttleworthia*, *Megasphaera and Gemella* were discriminant for WLWH with HPV positive while *Veillonella* was discriminant for WLWH without HR-HPV. *Veillonella* was also found to discriminate women without HPV from women with HPV infection irrespective of their HIV status. This latter result is in concordance with a recent study by Dareng et al. that found overrepresentation of *Veillonella* (*V. montpellierensis*) in HIV-negative women who were HR-HPV negative [21]. *Veillonella* has been found in women with bacterial vaginosis [45], in women with abnormal pH [39] but also in asymptomatic healthy women [46]. At species level, we identified *V. montpellierensis*, which has been shown to contribute to biofilm formation [47]. The exact role of *Veillonella* is currently unknown, and might dependent on the presence of other bacterial species [21]. Interestingly, *Shuttleworthia* together with *Prevotella* and Streptococcaceae were related to HSIL [48]. Also, *Megasphaera* has been linked to increased risk of HSIL and cervical cancer in women with HR-HPV[49]. Larger longitudinal cohorts are needed, not only to decipher the complex interplay between the VM, HIV and HPV infection, but also to understand how ART impacts this interplay. This could have important implications for the clinical management of WLWH [22, 26, 27]. The ultimate goal is to improve interventions aiming at reducing the burden of HPV and the global risk of cervical cancer in this vulnerable at-risk population. More studies are also needed to elucidate if the VM and its different CST types might be relevant and useful markers for the clinical management of HPV infection and colpocytological abnormalities and whether therapeutics should be tailored according to different VM compositions (personalized screening and clinical management based on the VM).

To our surprise, we found no correlation between vaginal pH and *Lactobacillus* spp. and between genital soluble markers of inflammation and the top 20 genera. The lack of correlation between vaginal pH and *Lactobacillus* spp. might reflect the limitation of using 16S sequencing and our primer selection (V3V4). The absence of correlation between vaginal inflammation and the top 20 genera might be explained by the method of collection used; we used the supernatant from the cervical cytobrush while most studies use cervicovaginal lavages (CVL) [7, 50, 51]. Techniques for the collection of female genital tract secretions are important [52] and CVL has been shown to be a superior method for cytokine assay reproducibility [53]. However, collection of CVL was not possible in our study. Also, we found no evidence of increased genital inflammation in WLWH and SNW with HPV infection compared to WLWH and SNW without HPV infection in concordance with Shannon and colleagues [54] but in contrast with Liebenberg and colleagues [50], these differences could be attributed to the fact that our cohort of WLWH were on ART for several years, therefore these were not women at risk of HIV acquisition. We also did not find an association between Lactobacillus and HR-HPV infection as reported in other studies. We did however find that WLWH with HPV infection had the highest level of IL-6 and CD8 + immune activation compared with SNW without HPV infection, reflecting a generalized state of chronic inflammation, which has been shown to be a risk factor for cancer development [55].

We realize that our study has several limitations. First, it is a relatively small cohort, which reduced the statistical power to detected differences between study groups. We lacked information on whether women who participated in this study had bacterial vaginosis (BV), women were neither diagnosed with BV using the Amsel´s criteria nor the Nugent scoring. Information on some co-variates was also missing, such as the menstrual cycle time point, and our information on sexual practices was incomplete. Also, cervical cytobrush sampling did not permit us to perform flow cytometry because of too low cellular yield. We also are aware that the use of the V3-V4 16S region is best suited for genus-level identification and species-level identification of *Lactobacillus* might have been hampered by our primer selection.

## Conclusion

In summary, the predominance of *L. iners* in the VM, combined with the high prevalence of HR-HPV has potential clinical implications for WLWH and SNW alike. Therefore, WLWH could benefit from closer follow-ups and might be candidates for microbiome-based interventions. Further work is warranted to understand the complex interactions between the host, the VM and HPV in women co-infected with HIV and HR-HPV, including WLWH on ART.

## Methods

### Ethics statement

This research protocol was approved by the Ethics and the Ethics in Research Committees of the National Institute of Respiratory Diseases (INER). All participants were adults (over 18 years old) and gave written informed consent in accordance with the Declaration of Helsinki. Both sample collection and laboratory testing were conducted at the Centre for Research in Infectious Diseases (CIENI) of the National Institute for Respiratory Diseases (INER).

### Subject enrollment

We enrolled 83 women between April 2016 and January 2017 in a cross-sectional study. Study participants were grouped according to their HIV status: HIV-uninfected (SNW, *n* = 39) and HIV-infected (WLWH, *n* = 44). WLWH were enrolled by convenience as they were already taking part in an HPV surveillance protocol and periodically attended the CIENI clinic (every 6 months to a year for follow-up HPV testing) and were aware of their HIV status. SNW were enrolled by direct advertising for study subjects (recruitment posters, word to mouth and/or oral communication). All women were given a structured questionnaire covering sociodemographic characteristics and sexual behaviors by trained female physicians.

### Plasma viral load and CD4 + T cell counts

HIV status was confirmed by two consecutive determinations using the VIDAS® HIV Duo Ultra (VIDAS, BioMérieux) and the Genscreen™ Ultra HIV Ag-Ab enzyme immunoassay (Evolis, Bio-Rad). None of the SWN taking part in this study tested positive for HIV. HIV plasma viral load was determined in plasma by automated real-time polymerase chain reaction (PCR) using the m2000 system (Abbott, Abbott Park, IL, USA) with a detection limit of 40 HIV-1 RNA copies/mL. Lymphocyte populations: CD45, CD3, CD4 and CD8 (absolute numbers and %) and CD4/CD8 ratio were obtained by flow cytometry using Multitest CD45/CD4/CD8/CD3 kit in FACScalibur instruments (BD Biosciences, San Jose, CA).

### HPV detection

Women underwent a pelvic examination performed by healthcare professionals from CIENI, INER. First, vaginal pH was measured by inserting a sterile cotton swab (Dacron Swab) into the vagina. The moisten cotton swab was then applied onto a pH-indicator strip (N-Labstix, Siemens, Erlangen, Germany). The pH number was determined by comparing the color of the pH strip to the color chart and recorded. Next, cervical and vaginal samples were collected for cervical cytology and HPV testing, respectively. Cervical cytology was assessed using conventional cytology and reported according to the Bethesda classification. HPV testing was performed by real-time PCR using the Abbott Real-time High-Risk HPV assay on the m2000 system (Abbott, Abbott Park, IL, USA), which detects HPV16, HPV18 and 12 other high-risk HPV genotypes (31, 33, 35, 39, 45, 51, 52, 56, 58, 59, 66 and 68).

### Sample collection

Research samples were collected by trained healthcare professionals and included one cervical cytobrush (Cervex Brush) and three mid-vagina swabs (Dacron Swab). Eighteen mL of peripheral blood were also collected at the time of pelvic examination in ethylenediaminetetraacetic acid (EDTA) tubes by venous puncture. Research samples (cervical cytobrush and vaginal swabs) were kept on ice and transported to the laboratory within 30 min of sampling and were either immediately processed (cervical cytobrush, EDTA blood) or kept at -80 °C (mid-vaginal swabs) until needed.

### Sample processing

Peripheral blood (18 mL) was immediately processed by centrifugation. Plasma samples were immediately frozen at -80 °C until use. Peripheral blood mononuclear cells (PBMCs) were purified from whole blood by density gradient centrifugation using lymphoprep (Axis-Shield, Oslo, Norway) according to the manufacturer´s instructions. PBMCs were cryopreserved in 90% fetal bovine serum (FBS, Gibco by Life Technologies, Washington, DC, USA) and 10% dimethyl sulfoxide (DMSO, Sigma-Aldrich, California, USA). Cervical cytobrushes were placed in 15-ml conical tubes containing 2 mL of RPMI 1640 medium, immediately placed on ice and transported to the laboratory within 30 min of sampling. Cervical cells were removed from the cytobrush by shaking, further washing away cells from the cytobrush by adding 1 mL of RPMI 1640 medium directly onto the cytobrush. Cervical cells were recovered by centrifugation at 1,900 rpm for 6 min. The cytobrush supernatant was aliquoted into 3 separate tubes containing 1 mL each and immediately frozen at -80 °C. The number of recovered cervical cells were too few for flow cytometry, so they were not used.

### DNA extraction from vaginal swabs

Bacterial DNA was extracted from the first vaginal swab using the PureLink Microbiome DNA purification kit (ThermoFisher Scientific, Carlsbad, CA, USA) according to the manufacturer´s instructions. Briefly, vaginal swabs were transferred from the -80 °C to the laboratory on dry ice and then placed in a 1.5 mL Eppendorf low-bind tube containing 800 µl of S1-Lysis Buffer. 100 µl of S2-Lysis Enhancer was added to each tube and vortexed, followed by a 10 min incubation at 65 °C and bead beat for 10 min on a vortex with a horizontal adapter. Swabs were not removed during the incubation and bead beating steps to maximize DNA extraction. Tubes were centrifuged for 1 min at 14,000 × *g*. Five hundred µl of the supernatant was transferred to a new tube and 900 µl of the S4-Binding buffer was added. After, the entire mix was loaded on to a spin column-tube assembly (2 × 700 µl) and centrifuged at 14,000 × *g* for 1 min. The spin column was washed by centrifugation with 500 µl of S5-Wash buffer and total DNA was eluted in 50 µl of S6-Elution buffer. DNA purity and quality were assessed by absorbance on a Nanodrop N1000 (ThermoFisher Scientific, Carlsbad, CA, USA) by measuring the A260/A280 ratio.

### 16S rRNA targeted sequencing, processing and analysis

For each DNA sample, a 16S amplicon library was prepared as instructed in the 16S Metagenomic Sequencing Library Preparation (Illumina, San Diego, CA, USA) with minor modifications. PCR amplification was performed using the published primers for the V3–V4 regions: forward, 5’-CCTACGGGNGGCWGCAG-3´; and reverse, 5’- GACTACHVGGGTATCTAATCC-3´[56]. The V3-V4 region of the 16S rRNA gene was amplified by triplicate polymerase chain reactions (PCR). Triplicate PCR reactions were pooled per sample and purified using AgenCourt AMPure XP beads (Beckman Dickson, Atlanta, Georgia). Dual indices were attached using the Nextera XT Index Kit (Illumina). The index PCR clean-up step was repeated to ensure complete purification of the index libraries before quantification, normalization and pooling. Libraries were paired-end sequenced 2 × 300 cycles on the Illumina MiSeq™ platform with a final concentration of 14 pM with 25% of PhiX. Controls for each DNA extraction and library preparation batch were done in parallel to account for reagents contamination. We used Qiime2 (Quantitative Insights into Microbial Ecology 2, version 2019.4) to extract high-quality sequences [57]. Selection of amplicon variant sequences (ASVs) was performed using dada2. A total number of 1,422 ASVs were identified, and a median of 172,046 (IQR = 113,868.5–240,004.5) sequences were retained after dada2. Taxonomy was assigned using the q2‐feature‐classifier using the classify‐sklearn naïve Bayes taxonomy classifier against the SILVA 99% database reference sequences (release 132) and trained to the V3-V4 region of the 16S rRNA gene. Phylogenetic tree was constructed with fasttree2 (via q2‐phylogeny) after aligning all ASVs with mafft. Artifacts generated with Qiime2 were imported into R software (version 4.0.5) through Phyloseq package for further manipulation and graph visualization. Rarefaction was performed at a sampling depth of 36,034 sequences/sample (corresponding to 90% of the sample with the least sequences). Principal Coordinate analyses (PCoA) were used to visualize microbial communities and permutational multivariate analysis of variance (PERMANOVA) was used to assess differences in beta diversity (default parameters: 999 permutations). We used Bray–Curtis dissimilarity for beta diversity. To identify significant differences in microbial composition (taxa) between groups, we performed linear discriminant analysis effect size (LEfSe) with an LDA threshold 3 or higher, and alpha value for factorial Kruskal–Wallis test 0.05, alpha value for pairwise Wilcoxon test 0.05 [58]. Prior, ASVs not seen more than 5 times in at least 5% of samples were removed, to avoid including ASVs with small mean and outliers. Spearman correlations were performed using rcorr() function and visualized using corrplot() function.

### Flow cytometry

Cryopreserved PBMC were thawed, washed, stained and batched tested. Activated CD4 and CD8 T cells were identified using the following extracellular staining panel: BV570-CD3 (clone: UCHT1), BV605-CD8 (clone: RPA-T8), BV711-CD38 (clone: HIT2) and BV785-HLADR (clone: UCHT1) from Biolegend (San Diego, CA, USA), PE-Cy5.5-CD4 (clone: MHCD0418) and LIVE/DEAD™ Fixable Aqua Dead Cell Stain Kit from Invitrogen (ThermoFisher scientific, CA). Staining was acquired on an LSR Fortessa FACS cytometer (Becton Dickinson Biosciences, San Jose, CA, USA). Quality control was performed for each experiment using BD cytometer Setup & Tracking Beads (BD biosciences, San Jose, California, USA). Individually stained beads (BD Comp Beads, eBiosciences, San Diego, CA, USA) were used as compensation controls. FMO (fluorescence minus one) was used as gating control. Data analysis was performed with FlowJo V10 software (Tree Star, Ashland, OR, USA). FlowAI was used to perform quality control of all FCS files. In total, the median number of recorded events was 185,909 [118026–302792] per sample. The gating strategy is shown in Additional file 14.

### Quantification of soluble markers by multiplex bead array kit

Concentrations of 19 cytokines (G-CSF, GM-CSF, IFN-α, IFN-γ, IL-1β, IL-1RA, IL-2, IL-2R, IL-4, IL-5, IL-6, IL-7, IL-8, IL-10, IL-12 (p40/p70), IL-13, IL-15, IL-17 and TNF-α), 7 chemokines (Eotaxin, IP-10, MCP-1, MIG, MIP-1α, MIP-1β and RANTES) and 4 growth factors (EGF, FGF-basic, HGF and VEGF) were determined in cytobrushes´s supernatants and plasma samples using the Human cytokine Magnetic 30-Plex Panel (ThermoFisher Scientific, Vienna, Austria), read on Luminex™ 100™/200™ detection system and analyzed on xPONENT™ software (ThermoFisher Scientific, Massachusetts, USA). All standards and samples were run in duplicates. Plasma and cytobrush supernatant samples were diluted 1:2. All procedures were performed by the same operator according to the manufacturers´ instructions. Lyophilized standards were reconstituted as instructed and diluted at serial concentrations for the generation standard curves. A 5-parameter logistic regression formula was used to calculate sample concentrations from the standard curve (pg/mL). To be included in subsequent analysis, the following criteria was used for each analyte: quantified within the detection limits (above the lower and below the higher detection limit) in more than 60% of samples, values that were extrapolated beyond the standard curve were assigned the determined value. Concentrations of growth factors, chemokines and cytokines were log_10_-transformed (verified using the Shapiro–Wilk normality test).

### Statistical analysis

Demographic and behavioral data were reported using descriptive statistics. Wilcoxon Rank Sum test was used to compare continuous variables and chi square test or Fisher's exact test for categorical variables. Kruskal–Wallis test was used to compare more than 2 groups, *p* values were adjusted for multiple comparisons using Dunn´s multiple comparison test. Statistical analyses were performed using STATA/SE 14 (STATA Corp. LP, College Station, Texas, USA) and GraphPad Prism v8 (San Diego, California, USA). Correlation coefficient (rho) and *p* values were obtained using the Spearman’s correlation test. Logistic regression analyses were conducted to identify risk factors association with HPV infection (predictors: age, smoking, age at first sexual intercourse, number of total sexual partners, vaginal pH, HIV, parity, condom use, previous history of STD, cervical cytology abnormalities, relative abundance of *Lactobacillus*). For all analyses, *p* values < 0.05 were considered statistically significant.

## Supplementary Information

**Additional file 1.** Data on reproductive health and sexual practices.

**Additional file 2.** HIV clinical parameters in WLWH (*n*=44).

**Additional file 3.** Risk factors associated with HPV infection.

**Additional file 4.** Log_10_ concentration of cytokines, chemokines and growth factors in cytobrush supernatant stratified by HIV or HPV status.

**Additional file 5.** Log_10_ Concentration (pg/mL) of plasma cytokines, chemokines and growth factors stratified by HIV or HPV status.

**Additional file 6.** Log_10_ plasma IL-6 concentration (pg/mL) stratified by HIV and HPV status.

**Additional file 7.** Frequencies of HLADR+ CD38+ CD4+ and CD8+ T cells stratified by HIV or HPV status.

**Additional file 8.** Frequencies of HLADR+ CD38+ CD4+ and CD8+ T cells stratified by HIV and HPV status.

**Additional file 9.** Alpha diversity is neither different by HIV status nor HPV status.

**Additional file 10.** Beta diversity is neither different by HIV status nor HPV status.

**Additional file 11.** Taxonomic profile of the vaginal microbiota at genus level in seronegative women and women living with HIV with or without HPV infection.

**Additional file 12.** The vaginal microbiota of women living with HIV and seronegative women with or without HR-HPV infection at species level (top 20 species).

**Additional file 13.** Inverse correlations were found between *Lactobacillus* and *Gardnerella*, *Prevotella*, *Dialister, *and *Atopobium*.

**Additional file 14.** Representative gating strategy.

## Data Availability

16S rRNA sequences are available at the Sequence Read Archive (SRA) under BioProject PRJNA814210. All other datasets used and analyzed during the current study are available from the corresponding author on reasonable request.
