# Supplementary material for: The vaginal microbiota of women living with HIV on suppressive antiretroviral therapy and its relation to high-risk human papillomavirus infection
Source: BMC Microbiol. 2023 Jan 19;23:21. doi: 10.1186/s12866-023-02769-1 (PMC9850673; doi:10.1186/s12866-023-02769-1)
Supplement: Supplementary file 1 — Additional file 1. Data on reproductive health and sexual practices. [file 12866_2023_2769_MOESM1_ESM.docx]

**Additional file** **1. Data on reproductive health and sexual practices**

|  | **All** | **SNW** | **WLWH** | **P value** |
| --- | --- | --- | --- | --- |
| **Number** | 83 | 39 | 44 |  |
| **Number of children**  Median (min, max) | 2 (0-8) | 1 (0-6) | 2 (0-8) | 0.0009* |
| **Number of parities**  Median (min, max) | 2 (0-8) | 2 (0-7) | 2 (0-8) | 0.0045* |
| **Anal sex**  No  Yes  Missing data | 21 (53.85)  18 (46.15)  44 | 21 (53.85)  18 (46.15)  0 | 0 (0)  0 (0)  44 | NA |
| **Oral sex**  No  Yes  Missing data | 37 48.05)  40 (51.95)  6 | 12 (30.77)  27 (69.23)  0 | 25 (56.79)  13 (34.21)  6 | 0.003* |
| **Oro-anal sex**  No  Yes  Missing data | 72 (94.74)  4 (5.26)  7 | 35 (92.11)  3 (7.89)  1 | 37 (97.37)  1 (2.63)  6 | 0.614 |
| **Previous PAP**  No  Yes | 6 (7.23)  77 (92.77) | 2 (5.13)  37 (94.87) | 4 (9.09)  40 (90.91) | 0.679 |
| **Last year PAP**  No  Yes  Missing data | 55 (68.75)  25 (31.25)  3 | 20 (51.28)  19 (48.72)  0 | 35 (85.37)  6 (14.63)  3 | 0.001* |
| **Presence of gynecological symptoms**  No  Yes | 34 (40.96)  49 (59.04) | 13 (33.33)  26 (66.67) | 21 (47.73)  23 (52.27) | 0.263 |
| **Intermenstrual bleeding**  No  Yes  Missing data | 68 (82.93)  14 (17.07)  1 | 30 (76.92)  9 (23.08)  0 | 38 (88.37)  5 (11.63)  1 | 0.241 |
| **Vaginal discharge**  No  Yes | 46 (55.42)  37 (44.58) | 19 (48.72)  20 (51.28) | 27 (61.36)  17 (38.64) | 0.275 |
| **Dyspareunia**  No  Yes  Missing data | 55 (73.33)  20 (26.67)  8 | 26 (68.42)  12 (31.58)  1 | 29 (78.38)  8 (21.62)  7 | 0.435 |
| **Pelvic pain**  No  Yes | 58 (69.88)  25 (30.12) | 24 (61.54)  15 (38.46) | 34 (77.27)  10 (22.73) | 0.152 |
| **Postcoital bleeding**  No  Yes  Missing data | 67 (89.33)  8 (10.67)  8 | 31 (81.58)  7 (18.42)  1 | 36 (97.30)  1 (2.70NA)  7 | 0.056* |

Data is expressed as n (%), unless stated otherwise. Wilcoxon Rank Sum test was used to compare continuous variables and chi2 test or Fisher's exact test for categorical variables. * p<0.05 (statistical significance). Abbreviations: max: maximum, min: minimum, PAP: Papanicolaou, NA: not applicable, SNW: Seronegative women, WLWH: Women living with HIV
