# Supplementary material for: The vaginal microbiota of women living with HIV on suppressive antiretroviral therapy and its relation to high-risk human papillomavirus infection
Source: BMC Microbiol. 2023 Jan 19;23:21. doi: 10.1186/s12866-023-02769-1 (PMC9850673; doi:10.1186/s12866-023-02769-1)
Supplement: Supplementary file 2 — Additional file 2. HIV clinical parameters in WLWH (n=44). [file 12866_2023_2769_MOESM2_ESM.docx]

**Additional file** **2. HIV clinical parameters in WLWH (n=44)**

|  | **WLWH** |
| --- | --- |
| **Number** | 44 |
| **ART regimen**  NNRTI  PI  INSTI  None^¥^ | 24 (54.55)  12 (27.27)  4 (9.09)  4 (9.09) |
| **Nadir CD4+ T-cell count (cells/mm^3^)** | 105.5 [29.25-240.8] |
| **Stratification by Nadir CD4+ T-cell count**  CD4<200  CD4>200 | 29 (65.91)  15 (34.09) |
| **CD4+ T-cell count (cells/mm^3^)** | 529.5 [307.3-728.8] |
| **Stratification by CD4+ T-cell count**  CD4<200  200<CD4<500  CD4>500 | 4 (9.09)  17 (38.64)  23 (52.27) |
| **CD4+ %** | 26.5 [19.25-34] |
| **CD8+ T-cell count (cells/mm^3^)** | 722 [508.5-952] |
| **CD8+ %** | 39 [29.25-50] |
| **CD4+/CD8+ ratio** | 0.7 [0.415-1.208] |
| **HIV-1 RNA VL (copies/mL)** | 39 [39-39] |
| **HIV-1 RNA pVL (Log copies/mL)** | 1.6 [1.6-1.6] |

Data expressed as n (%) or median [IQR: interquartile range].

^¥^None refers to the women who are elite controllers (defined as WLWH with undetectable HIV-1 viral load in the absence of ART for more than 1 year).

Abbreviations: HIV-1: Human immunodeficiency virus type 1, INSTI: Integrase Strand Transfer Inhibitors, mm^3^: cubic millimeter, NNRTI: Non-nucleoside reverse transcriptase inhibitors, PI: Protease inhibitors, pVL: log plasma viral load, RNA: Ribonucleic acid, SNW: Seronegative women, VL: viral load, WLWH: Women living with HIV
