# Supplementary material for: The vaginal microbiota of women living with HIV on suppressive antiretroviral therapy and its relation to high-risk human papillomavirus infection
Source: BMC Microbiol. 2023 Jan 19;23:21. doi: 10.1186/s12866-023-02769-1 (PMC9850673; doi:10.1186/s12866-023-02769-1)
Supplement: Supplementary file 3 — Additional file 3. Risk factors associated with HPV infection. [file 12866_2023_2769_MOESM3_ESM.docx]

**Additional file 3. Risk factors associated with HPV infection**

|  | **HPV negative** | **HPV positive** | **P value** |
| --- | --- | --- | --- |
| **Number of women** | 57 | 26 |  |
| **Age (years), median (min, max)** | 43 (23-65) | 41 (21-69) | 0.2547 |
| **Civil Status, n (%)**  Single  Free union  Married  Divorced  Widowed  Missing data | 15 (26.79)  14 (25.00)  11 (16.64)  13 (23.21)  3 (5.36)  1 | 9 (34.62)  7 (26.92)  5 (19.23)  3 (11.54)  2 (7.69)  0 | 0.772 |
| **Level of education, n (%)**  Illiterate  Primary education  Secondary education | 2 (3.51)  15 (26.32)  40 (70.18) | 1 (3.85)  8 (30.77)  17 (65.38) | 0.905 |
| **Occupation, n (%)**  Employed  Unemployed  Student  Informal Employment | 17 (29.82)  1 (1.75)  5 (8.77)  34 (59.65) | 9 (34.62)  1 (3.85)  1 (3.85)  15 (57.69) | 0.749 |
| **Smoking, n (%)**  Actual  Never  Used to smoke | 6 (10.53)  39 (68.42)  12 (21.05) | 4 (15.38)  15 (57.69)  7 (26.92) | 0.597 |
| **Alcohol usage, n (%)**  Light  Moderate  Missing data | 49 (89.09)  6 (10.91)  2 | 24 (92.31)  2 (7.69)  0 | 0.772 |
| **Age at sexual debut (years)**, **median (min, max)** | 17 (6-36) | 17 (13-26) | 0.8474 |
| **Total Nb of sexual partners, median (min, max)** | 3 (1-20) | 3 (1-15) | 0.6814 |
| **Nb of sexual partners in the last year, mean (min, max)** | 1 (0-4) | 1 (0-4) | 0.8516 |
| **Nb of children, median (min, max)** | 2 (0-6) | 2 (0-8) | 0.9502 |
| **Nb of pregnancies, median (min, max)** | 2 (0-7) | 2 (0-8) | 0.4039 |
| **Anal sex, n (%)**  NO  YES  Missing data | 14 (40)  12 (60)  31 | 7 (53.85)  6 (46.15)  13 | >0.999 |
| **Oral sex, n (%)**  NO  YES  Missing data | 24 (45.28)  29 (54.72)  4 | 13 (54.17)  11 (45.83)  2 | 0.623 |
| **Oro-anal sex, n (%)**  NO  YES  Missing data | 49 (94.23)  3 (5.77)  5 | 23 (95.83)  1 (4.17)  2 | >0.999 |
| **Condom use, n (%)**  Always  Sometimes  Never | 9 (15.79)  31 (54.39)  17 (29.82) | 3 (11.54)  14 (53.85)  9 (34.62) | 0.897 |
| **pH, median (min, max)** | 5.5 (5-9) | 5.5 (5-7) | 0.7481 |
| **Previous STD, n (%)**  NO  YES | 45 (78.95)  12 (21.05) | 19 (73.08)  7 (26.92) | 0.555 |
| **STD, n (%)**  Candidiasis  Chlamydia  Genital herpes  Genital warts  Syphilis  Others | 20 (35.08)  2 (3.5)  1 (1.75)  6 (10.52)  1 (1.75)  5 (8.77) | 11 (42.3)  1 (3.84)  3 (11.53)  1 (3.84)  0 (0)  3 (11.53) | 0.4551 |
| **Menopause, n (%)**  NO  YES  Missing data | 45 (80.36)  11 (19.64)  1 | 23 (88.46)  3 (11.54)  0 | 0.531 |
| **Previous PAP, n (%)**  NO  YES | 5 (8.77)  52 (91.23) | 1 (3.85)  25 (96.15) | 0.422 |
| **Last year PAP, n (%)**  NO  YES  Missing data | 38 (69.09)  17 (30.31)  2 | 17 (68.00)  8 (32)  1 | >0.999 |
| **Presence of gynecological symptoms, n (%)**  NO  YES | 22 (38.6)  35 (61.4) | 12 (46.15)  14 (53.85) | 0.516 |
| **Intermenstrual bleeding, n (%)**  NO  YES  Missing data | 46 (82.14)  10 (17.86)  1 | 22 (84.62)  4 (15.38)  0 | >0.999 |
| **Vaginal discharge, n (%)**  NO  YES | 33 (57.89)  24 (42.11) | 13 (50)  13 (50) | 0.502 |
| **Dyspareunia, n (%)**  NO  YES  Missing data | 39 (73.58)  14 (26.42)  4 | 16 (72.73)  6 (27.27)  4 | >0.999 |
| **Pelvic pain, n (%)**  NO  YES | 40 (70.18)  17 (29.82) | 18 (69.23)  8 (30.77) | 0.931 |
| **Postcoital bleeding, n (%)**  NO  YES  Missing data | 47 (88.68)  6 (26.42)  4 | 20 (72.73)  2 (27.27)  4 | >0.999 |
| **HPV vaccine, n (%)**  NO  YES  Missing data | 29 (90.63)  3 (9.38)  25 | 14 (87.50)  2 (12.50)  10 | >0.999 |
| **PAP Result, n (%)**  Normal  Abnormal (Dysplasia + Inflammation)  Missing data | 36 (76.60)  11 (23.40)  10 | 18 (78.26)  5 (21.74)  3 | >0.999 |
| **Cervical cytology**  NIML  Abnormal (LSIL + HSIL)  Missing data | 44 (89.80)  5 (10.20)  8 | 22 (91.67)  2 (8.33)  2 | >0.999 |
| **HIV status, n (%)**  Negative  Positive | 26 (45.61)  31 (54.39) | 13 (50.00)  13 (50.00) | 0.710 |
| **ART use (WLWH), n (%)**  YES  NO | 28 (49.12)  3 (5.26) | 12 (46.15)  1 (3.85) | 0.914 |
| **ART regimen (WLWH), n (%)**  NNRTI  PI  INSTI | 17 (60.71)  8 (28.57)  3 (10.71) | 7 (58.33)  4 (33.33)  1 (8.33) | 0.942 |
| **Nadir CD4+ T-cell count (WLWH), n (%)**  CD4<200  CD4>200 | 20 (35.09)  11 (19.3) | 9 (34.62)  4 (15.38) | >0.999 |

Data expressed as n (%) or median (min-max)

Wilcoxon Rank Sum test was used to compare continuous variables and chi2 test or Fisher's exact test for categorical variables. p<0.05 (statistical significance)

Abbreviations: ART: antiretroviral therapy, LSIL: low-grade squamous intraepithelial lesions, HIV: human immunodeficiency virus, INSTI: integrase strand transfer inhibitor, HPV: human papillomavirus, HSIL: high-grade squamous intraepithelial lesions, Nb: number, NILM: negative for intraepithelial lesion or malignancy NNRTI: non-nucleoside reverse transcriptase inhibitor, PAP: Papanicolaou, PI: protease inhibitors, STD: Sexually Transmitted Diseases
