# Supplementary material for: The vaginal microbiota of women living with HIV on suppressive antiretroviral therapy and its relation to high-risk human papillomavirus infection
Source: BMC Microbiol. 2023 Jan 19;23:21. doi: 10.1186/s12866-023-02769-1 (PMC9850673; doi:10.1186/s12866-023-02769-1)
Supplement: Supplementary file 4 — Additional file 4. Log10 concentration of cytokines, chemokines and growth factors in cytobrush supernatant stratified by HIV or HPV status. [file 12866_2023_2769_MOESM4_ESM.docx]

**Additional file 4. Log_10_ concentration of cytokines, chemokines and growth factors in cytobrush supernatant stratified by HIV or HPV status**

|  | **HIV** | | | **HPV** | | |
| --- | --- | --- | --- | --- | --- | --- |
|  | **SNW** | **WLWH** | **P value** | **HPVN** | **HPVP** | **P value** |
| **Cytokines** | | | | | | |
| G-CSF | 2.24 [2.17-2.54] | 2.21 [2.16-2.46] | 0.5750 | 2.23 [2.17-2.54] | 2.21 [2.16-2.61] | 1 |
| IFN-α | 1.43 [1.32-1.48] | 1.42 [1.32-1.48] | 0.9797 | 1.44 [1.34-1.48] | 1.38 [1.32-1.48] | 0.2165 |
| IFN-γ | 1.18 [1.11-1.3] | 1.19 [1.11-1.27] | 0.6909 | 1.19 [1.12-1.29] | 1.14 [1.1-1.28] | 0.3441 |
| IL-1β | 1.35 [1.16-1.43] | 1.33 [1.17-1.41] | 0.7481 | 1.35 [1.18-1.43] | 1.24 [1.16-1.43] | 0.4728 |
| IL-2 | 0.65 [0.53-0.72] | 0.57 [0.20-0.7] | 0.3271 | 0.61 [0.20-0.7] | 0.61[0.53-0.78] | 0.2702 |
| IL-2R | 1.86 [1.7-1.92] | 1.82 [1.64-1.92] | 0.4036 | 1.85 [1.68-1.94] | 1.81 [1.66-1.9] | 0.3441 |
| IL-4 | 1.1 [1-1.21] | 1.12 [1.03-1.18] | 0.9026 | 1.12 [1.03-1.19] | 1.1 [0.99-1.25] | 0.9734 |
| IL-5 | 0.57 [0.4-0.64] | 0.61 [0.48-0.67] | 0.1434 | 0.59 [0.45-0.67] | 0.59 [0.39-0.62] | 0.2223 |
| IL-6 | 1.64 [1.5-1.83] | 1.63 [1.45-1.89] | 0.8544 | 1.67 [1.45-1.86] | 1.58 [1.5-1.9] | 0.6805 |
| IL-7 | 1.87 [1.84-1.92] | 1.88 [1.84-1.92] | 0.8720 | 1.9 [1.84-1.92] | 1.86 [1.81-1.9] | 0.1623 |
| IL-8 | 3.48 [2.99-4.17] | 3.44 [2.89-3.88] | 0.6984 | 3.44 [2.9-3.94] | 3.51 [2.74-4.17] | 0.9645 |
| IL-10 | 0.81 [0.72-0.95] | 0.78 [0.68-0.98] | 0.8905 | 0.82 [0.69-0.99] | 0.74 [0.64-0.86] | 0.3219 |
| IL-12 | 1.34 [1.22-1.4] | 1.28 [1.2-1.39] | 0.3177 | 1.31 [1.22-1.41] | 1.27 [1.2-1.36] | 0.1967 |
| IL-15 | 2.05 [1.97-2.12] | 2.07 [1.97-2.14] | 0.9026 | 2.07 [1.98-2.14] | 2.05 [1.95-2.11] | 0.2929 |
| IL-17 | 0.87 [0.65-1.01] | 0.86 [0.65-0.97] | 0.6780 | 0.88 [0.7-0.97] | 0.72[0.56-1.04] | 0.5518 |
| TNF-α | 0.74 [0.35-0.79] | 0.75 [0.27-0.79] | 0.8416 | 0.75 [0.31-0.79] | 0.74 [0.17-0.79] | 0.9522 |
| **Chemokines** | | | | | | |
| Eotaxin | 0.13 [-0.1-0.22] | 0.09 [-0.07-0.31] | 0.9269 | 0.12 [-0.03-0.26] | 0.1 [-0.16-0.22] | 0.6086 |
| IP-10 | 0.79 [0.67-1.15] | 0.81 [0.64-1.4] | 0.9067 | 0.86 [0.67-1.31] | 0.72 [0.64-1.14] | 0.3644 |
| MCP-1 | 2.3 [2.15-2.49] | 2.34 [2.18-2.48] | 0.7831 | 2.31 [2.13-2.44] | 2.3 [2.21-2.5] | 0.5932 |
| MIG | 1.95 [1.79-2.32] | 2.04 [1.86-2.4] | 0.8027 | 2 [1.79-2.41] | 1.98 [1.86-2.18] | 0.9114 |
| MIP-1α | 1.52 [1.44-1.65] | 1.55 [1.44-1.63] | 0.8344 | 1.52 [1.44-1.64] | 1.52 [1.45-1.72] | 0.8893 |
| MIP-1β | 1.68 [1.47-1.93] | 1.69 [1.44-2.05] | 0.7395 | 1.69 [1.42-2.04] | 1.66 [1.49-2.05] | 0.8952 |
| RANTES | 1.18 [1.08-1.37] | 1.18 [1.09-1.38] | 1 | 1.17 [1.04-1.39] | 1.18 [1.1-1.36] | 0.8674 |
| **Growth factors** | | | | | | |
| EGF | 1.36 [1.24-1.55] | 1.35 [1.26-1.5] | 0.5577 | 1.36 [1.26-1.52] | 1.34 [1.22-1.5] | 0.5590 |
| FGF basic | 0.99 [0.93-1.05] | 0.98 [0.92-1.05] | 0.7481 | 0.98 [0.93-1.05] | 0.99 [0.91-1.05] | 0.5741 |
| HGF | 1.9 [1.74-2] | 1.87 [1.72-2.03] | 0.5543 | 1.9 [1.72-2.02] | 1.82 [1.72-1.96] | 0.4832 |
| VEGF | 1.4 [1.34-1.44] | 1.39 [1.35-1.42] | 0.752 | 1.4 [1.35-1.44] | 1.37 [1.33-1.42] | 0.1891 |

Data expressed as median and IQR [interquartile range]. Wilcoxon Rank Sum test was used to compare groups. * p<0.05 (statistical significance)

Abbreviations: EGF: Epidermal Growth Factor, FGF basic: Fibroblast Growth Factor basic, G-CSF: Granulocyte-colony stimulating factor, HIV: Human immunodeficiency virus, HGF: Hepatocyte Growth Factor, HPVN: HPV negative, HPVP: HPV positive, IFN-α: Interferon-alpha, IFN-γ: Interferon-gamma, IL-1β: interleukin-1 beta, IL-2: Interleukin-2, IL-2R: Interkeukin-2 Receptor, IL-4: Interleukin-4, IL-5: Interleukin-5, IL-6: Interleukin-6, IL-7: Interleukin-7, IL-8: Interleukin-8, IL-10: Interleukin-10, IL-12: Interleukin-12, IL-15: Interleukin-15, IL-17: Interleukin-17, IP-10: Interferon-gamma-inducible Protein 10, MCP-1: Monocyte Chemoattractant Protein-1, MIG: Monokine Induced by Interferon-gamma, MIP-1α: Macrophage Inflammatory Protein 1 alpha, MIP-1β: Macrophage Inflammatory Protein 1 beta, pg/mL: picograms per milliliter, SNW: Seronegative women, TNF-α: Tumor Necrosis Factor alpha, VEGF: Vascular endothelial growth factor, WLWH: Women living with HIV.
