# Supplementary material for: The vaginal microbiota of women living with HIV on suppressive antiretroviral therapy and its relation to high-risk human papillomavirus infection
Source: BMC Microbiol. 2023 Jan 19;23:21. doi: 10.1186/s12866-023-02769-1 (PMC9850673; doi:10.1186/s12866-023-02769-1)
Supplement: Supplementary file 5 — Additional file 5. Log10 Concentration (pg/mL) of plasma cytokines, chemokines and growth factors stratified by HIV or HPV status. [file 12866_2023_2769_MOESM5_ESM.docx]

**Additional file 5. Log_10_ Concentration (pg/mL) of plasma cytokines, chemokines and growth factors stratified by HIV or HPV status**

|  | **HIV** | | | **HPV** | | |
| --- | --- | --- | --- | --- | --- | --- |
|  | **SNW** | **WLWH** | **P value** | **HPVN** | **HPVP** | **P value** |
| **Cytokines** | | | | | | |
| G-CSF | 2.23 [2.21-2.24] | 2.24 [2.22-2.26] | 0.2255 | 2.24 [2.19-2.25] | 2.24 [2.22-2.26] | 0.6566 |
| GM-CSF | 0.69 [0.68-0.72] | 0.7 [0.69-0.73] | 0.7261 | 0.69 [0.68-0.72] | 0.71 [0.69-0.75] | 0.2824 |
| IFN-γ | 1.34 [1.28-1.38] | 1.35 [1.3-1.38] | 0.4411 | 1.35 [1.28-1.38] | 1.35 [1.29-1.38] | 0.8971 |
| IL-1RA | 2.2 [2.08-2.41] | 2.27 [2.05-2.42] | 0.8649 | 2.27 [2.08-2.41] | 2.2 [2.02-2.4] | 0.5349 |
| IL-2R | 2.43 [2.24-2.63] | 2.52 [2.38-2.69] | 0.0596 | 2.52 [2.3-2.67] | 2.43 [2.27-2.58] | 0.4219 |
| IL-4 | 1.46 [1.45-1.48] | 1.47 [1.45-1.48] | 0.4558 | 1.46 [1.45-1.48] | 1.47 [1.46-1.53] | 0.2782 |
| IL-6 | 1.02 [0.98-1.07] | 1.05 [1.04-1.11] | 0.0164* | 1.04 [0.99-1.06] | 1.08 [1.04-1.10] | 0.037* |
| IL-8 | 0.81 [0.66-0.96] | 0.99 [0.86-1.11] | 0.0284* | 0.93 [0.82-1.03] | 0.8 [0.72-0.95] | 0.4381 |
| IL-12 | 2.38 [2.29-2.46] | 2.43 [2.32-2.54] | 0.0710 | 2.38 [2.3-2.5] | 2.43 [2.33-2.49] | 0.5907 |
| TNF-α | 1.02 [0.99-1.05] | 1.03 [1.01-1.05] | 0.5326 | 1.02 [1.01-1.05] | 1.01 [0.99-1.05] | 0.9112 |
| **Chemokines** | | | | | | |
| Eotaxin | 1.72 [1.57-1.83] | 1.88 [1.81-1.96] | <0.0001* | 1.83 [1.72-1.92] | 1.79 [1.61-1.94] | 0.4627 |
| IP-10 | 1.44 [1.26-1.56] | 1.69 [1.54-1.89] | <0.0001* | 1.56 [1.41-1.79] | 1.48 [1.36-1.65] | 0.2343 |
| MCP-1 | 2.38 [2.25-2.49] | 2.48 [2.36-2.59] | 0.0075* | 2.42 [2.3-2.53] | 2.44 [2.34-2.54] | 0.9341 |
| MIG | 2.24 [2.09-2.45] | 2.55 [2.29-2.75] | 0.0003* | 2.42 [2.18-2.64] | 2.35 [2.19-2.72] | 0.8160 |
| MIP-1β | 1.87 [1.68-1.99] | 1.88 [1.75-2.08] | 0.4785 | 1.88 [1.75-1.98] | 1.9 [1.72-2.05] | 0.9010 |
| **Growth factors** | | | | | | |
| EGF | 1.47 [1.37-1.64] | 1.54 [1.36-1.64] | 0.9775 | 1.51 [1.36-1.62] | 1.53 [1.36-1.64] | 0.8524 |
| HGF | 2.25 [2.05-2.32] | 2.31 [2.2-2.45] | 0.0275* | 2.26 [2.14-2.37] | 2.28 [2.2-2.37] | 0.7493 |

Data expressed as median [interquartile range]. Wilcoxon Rank Sum test was used to compare groups. * p<0.05 (statistical significance)

Abbreviations: EGF: Epidermal Growth Factor, G-CSF: Granulocyte-colony stimulating factor, GM-CSF: Granulocyte-Macrophage Colony-Stimulating Factor, HGF: Hepatocyte Growth Factor, HIV: human immunodeficiency virus, HPVN: HPV negative, HPVP: HPV positive, IFN-γ: Interferon-gamma, IL-1RA: Interleukin-1 Receptor Antagonist, IL-2R: Interleukin-2 Receptor, IL-4: Interleukin-4, IL-6: Interleukin-6, IL-8: Interleukin-8, IL-12: Interleukin-12, IP-10: Interferon-gamma-inducible Protein 10, MCP-1: Monocyte Chemoattractant Protein-1, MIG: Monokine Induced by Interferon-gamma, MIP-1β: Macrophage Inflammatory Protein 1 beta, pg/mL: picograms per milliliter, SNW: Seronegative women, TNF-α: Tumor Necrosis Factor alpha, WLWH: Women living with HIV.
