# Supplementary material for: The vaginal microbiota of women living with HIV on suppressive antiretroviral therapy and its relation to high-risk human papillomavirus infection
Source: BMC Microbiol. 2023 Jan 19;23:21. doi: 10.1186/s12866-023-02769-1 (PMC9850673; doi:10.1186/s12866-023-02769-1)
Supplement: Supplementary file 6 — Additional file 6. Log10 plasma IL-6 concentration (pg/mL) stratified by HIV and HPV status. [file 12866_2023_2769_MOESM6_ESM.docx]

**Additional file 6. Log_10_ plasma IL-6 concentration (pg/mL) stratified by HIV and HPV status**

|  | **SNW HPVN** | **SNW HPVP** | **WLWH HPVN** | **WLWH HPVP** | **KW p value** | **Adjusted p-values** |
| --- | --- | --- | --- | --- | --- | --- |
| **Cytokine** | | | | | | |
| IL-6 | 0.99 [0.97-1.04]  n=12 | 1.05 [1.02-1.08]  n=6 | 1.05 [1.01-1.09]  n=12 | 1.1 [1.05-1.24]  n=7 | 0.0207* | SNWmHPVN *vs* WLWH HPVP  p= 0.0127 |

Data expressed as median [interquartile range]. The number of samples are indicated for each group.

Kruskal-Wallis nonparametric test, followed by Dunn's multiple comparisons test (p-adjusted) was used to compare groups. Only significant adjusted p values are shown. * p<0.05 (statistical significance).

Abbreviations: HIV: Human immunodeficiency virus, HPVN: HPV negative, HPVP: HPV positive, IL-6: Interleukin-6, pg/mL: picograms per milliliter, SNW: Seronegative women, WLWH: Women living with HIV
