# Supplementary material for: The vaginal microbiota of women living with HIV on suppressive antiretroviral therapy and its relation to high-risk human papillomavirus infection
Source: BMC Microbiol. 2023 Jan 19;23:21. doi: 10.1186/s12866-023-02769-1 (PMC9850673; doi:10.1186/s12866-023-02769-1)
Supplement: Supplementary file 7 — Additional file 7. Frequencies of HLADR+ CD38+ CD4+ and CD8+ T cells stratified by HIV or HPV status. [file 12866_2023_2769_MOESM7_ESM.docx]

**Additional file 7. Frequencies of HLADR+ CD38+ CD4+ and CD8+ T cells stratified by HIV or HPV status**

|  | **HIV** | | | **HPV** | | |
| --- | --- | --- | --- | --- | --- | --- |
| **Frequency of T cell (%)** | **SNW** | **WLWH** | **P value** | **HPVN** | **HPVP** | **P value** |
| CD4+ HLADR+ CD38+ | 23.5 [17.6-26] | 10.4 [8.83-18.4] | <0.0001* | 16.7 [9.25-25.25] | 18.7 [14.6-26.8] | 0.1320 |
| CD8+ HLADR+ CD38+ | 8.77 [4.32-11.6] | 12.7 [8.48-19.4] | 0.0012* | 10.8 [6.34-14.2] | 12.9 [7.8-21.2] | 0.2926 |

Data is expressed as median and IQR [interquartile range]. Wilcoxon Rank Sum test was used to compare between groups. * p<0.05 (statistical significance).

PBMCs for 9 women were unavailable for immunophenotyping.

Abbreviations: CD: cluster of differentiation, HIV: Human immunodeficiency virus, HPV: Human papillomavirus, HPVN: HPV negative, HPVP: HPV positive, PBMCs: peripheral blood mononuclear cells, SNW: Seronegative women, WLWH: Women living with HIV.
