# Supplementary material for: The vaginal microbiota of women living with HIV on suppressive antiretroviral therapy and its relation to high-risk human papillomavirus infection
Source: BMC Microbiol. 2023 Jan 19;23:21. doi: 10.1186/s12866-023-02769-1 (PMC9850673; doi:10.1186/s12866-023-02769-1)
Supplement: Supplementary file 8 — Additional file 8. Frequencies of HLADR+ CD38+ CD4+ and CD8+ T cells stratified by HIV and HPV status. [file 12866_2023_2769_MOESM8_ESM.docx]

**Additional file 8. Frequencies of HLADR+ CD38+ CD4+ and CD8+ T cells stratified by HIV and HPV status**

| **Frequency of T cell (%)** | **SNW HPVN** | **SNW HPVP** | **WLWH HPVN** | **WLWH HPVP** | **KW p value** | **Adjusted p values** |
| --- | --- | --- | --- | --- | --- | --- |
| CD4+ HLADR+ CD38+ | 24.5  [16.5-25.8] | 22.5  [17.6-28] | 9.3  [7.37-16.9] | 16.2  [10.4-19.5] | <0.0001* | SNW/HPVN *vs* WLWH/HPVN  p= 0.0002  SNW/HPVP *vs* WLWH/HPVN  p= 0.0016 |
| CD8+ HLADR+ CD38+ | 9.07  [3.63-11.5] | 8.77  [4.51-13.6] | 12.4  [8.12-18.4] | 19.3  [8.75-30] | 0.0078* | SNW/HPVN *vs* WLWH/HPVP  p= 0.0176 |

Data expressed as a percentage (%) with median [interquartile range].

Kruskal-Wallis nonparametric test, followed by Dunn's multiple comparisons test (p-adjusted) was used to compare groups. * p<0.05 (statistical significance). Only significant adjusted p values are shown.

Abbreviations: CD: cluster of differentiation, HIV: Human immunodeficiency virus, HPV: Human papillomavirus, HPVN: HPV negative, HPVP: HPV positive, SNW: Seronegative women, WLWH: Women living with HIV.
