# Supplementary material for: The vaginal microbiota of women living with HIV on suppressive antiretroviral therapy and its relation to high-risk human papillomavirus infection
Source: BMC Microbiol. 2023 Jan 19;23:21. doi: 10.1186/s12866-023-02769-1 (PMC9850673; doi:10.1186/s12866-023-02769-1)
Supplement: Supplementary file 9 — Additional file 9. Alpha diversity is neither different by HIV status nor HPV status. [file 12866_2023_2769_MOESM9_ESM.docx]

**Additional file 9**: **Alpha diversity is neither different by HIV status nor HPV status.**


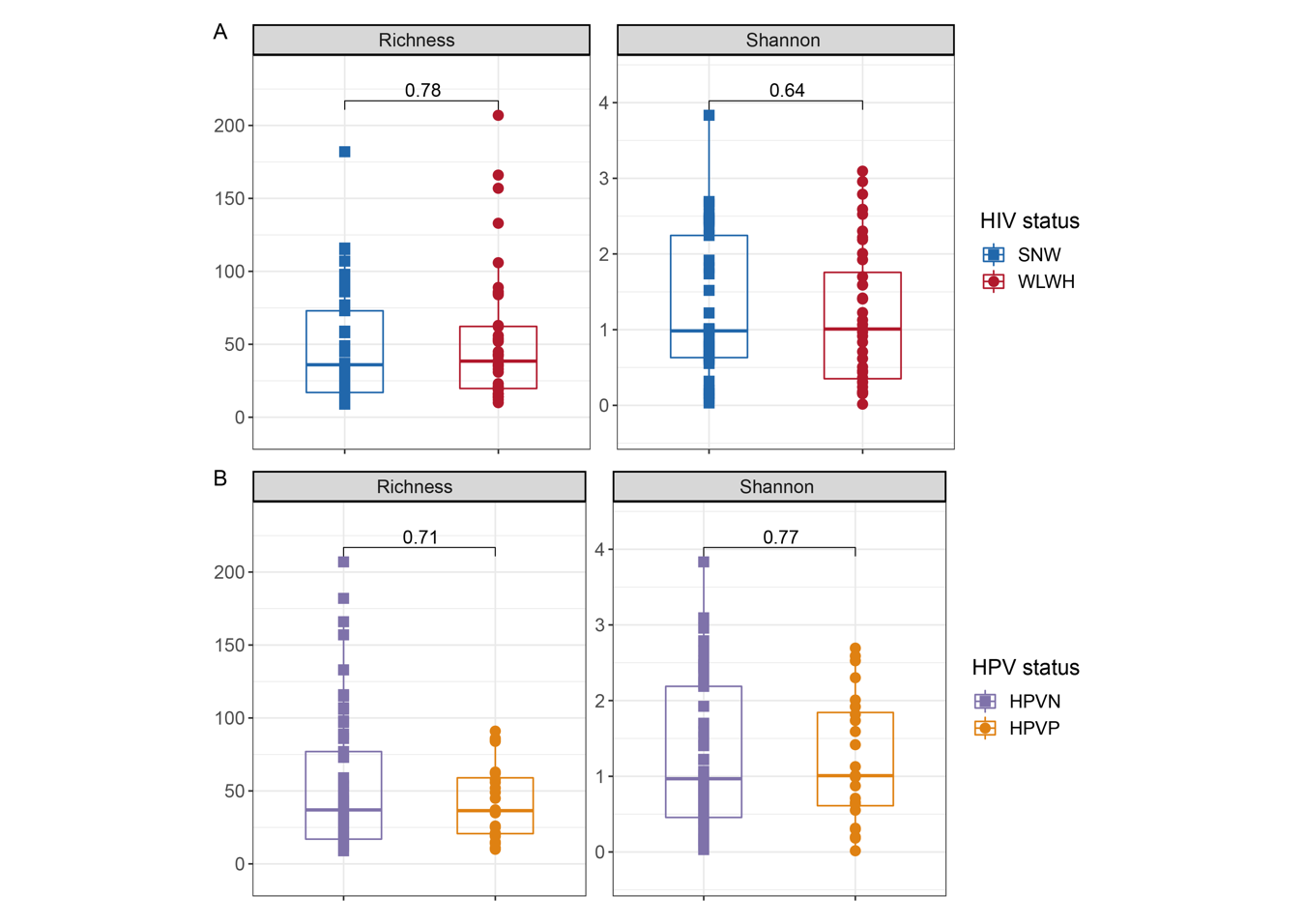


Legend:

Boxplots showing the median and interquartile range stratified by A. HIV status and B. HPV status. Two alpha diversity metrics were calculated: richness (number of observed species) and shannon (richness and evenness). Groups were compared using the Wilcoxon Rank Sum test.

Abbreviations: HIV: human immunodeficiency virus, HPV: human papillomavirus, HPVN: HPV negative, HPVP: HPV negative, SNW: seronegative women, WLWH: women living with HIV
