# Supplementary material for: The vaginal microbiota of women living with HIV on suppressive antiretroviral therapy and its relation to high-risk human papillomavirus infection
Source: BMC Microbiol. 2023 Jan 19;23:21. doi: 10.1186/s12866-023-02769-1 (PMC9850673; doi:10.1186/s12866-023-02769-1)
Supplement: Supplementary file 10 — Additional file 10. Beta diversity is neither different by HIV status nor HPV status. [file 12866_2023_2769_MOESM10_ESM.docx]

**Additional file 10**: **Beta diversity is neither different by HIV status nor HPV status.**


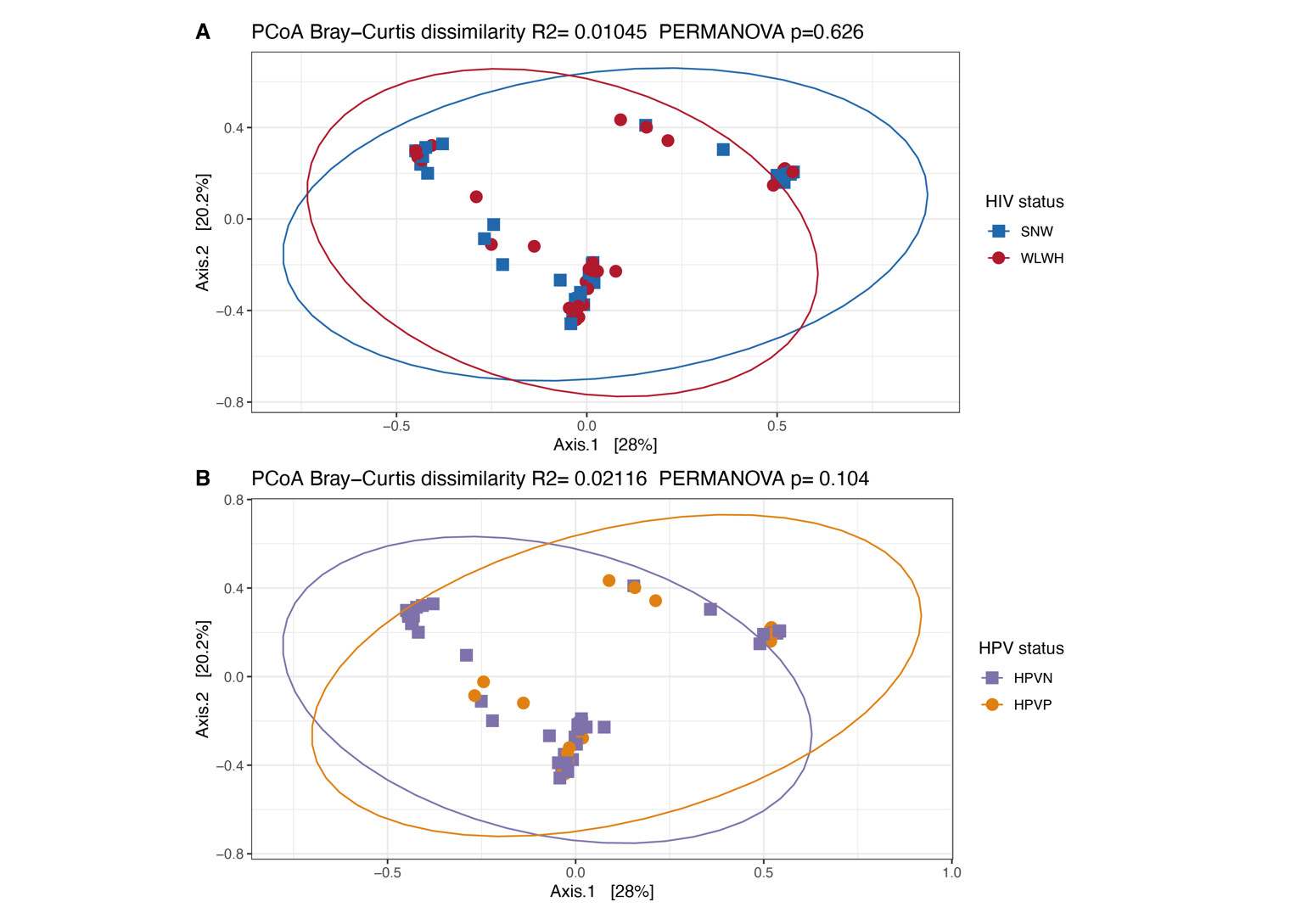


Legend:

Clustering of microbial communities was visualized using principal coordinate analysis (PCoA, Bray-Curtis dissimilarity) and differences were assessed by PERMANOVA after testing for homogeneity of dispersions (betadisper). We found no significant differences in microbial community structure when considering HIV status (R^2^=0.010, p=0.626), HPV status (R^2^= 0.021, p=0.104) or both (R^2^= 0.047, p= 0.235).

Abbreviations: HIV: human immunodeficiency virus, HPV: human papillomavirus, HPVN: HPV negative, HPVP: HPV negative, PERMANOVA: Permutational multivariate analysis of variance, R^2^: R squared, SNW: seronegative women, WLWH: women living with HIV
