# Supplementary material for: The vaginal microbiota of women living with HIV on suppressive antiretroviral therapy and its relation to high-risk human papillomavirus infection
Source: BMC Microbiol. 2023 Jan 19;23:21. doi: 10.1186/s12866-023-02769-1 (PMC9850673; doi:10.1186/s12866-023-02769-1)
Supplement: Supplementary file 11 — Additional file 11. Taxonomic profile of the vaginal microbiota at genus level in seronegative women and women living with HIV with or without HPV infection. [file 12866_2023_2769_MOESM11_ESM.docx]

**Additional file 11**: **Taxonomic profile of the vaginal microbiota at genus level in seronegative women and women living with HIV with or without HPV infection.**


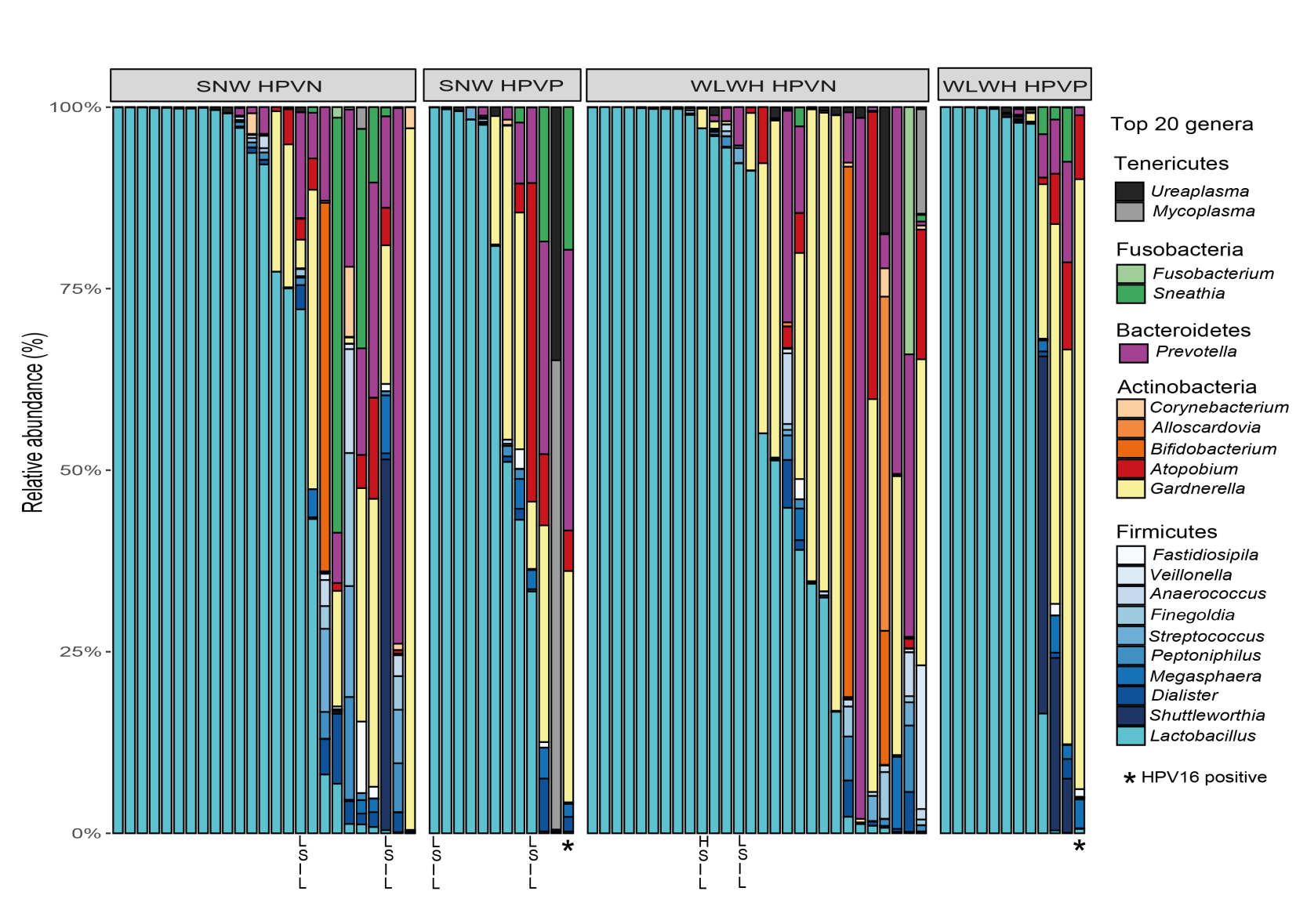


Legend:

Taxa barplots showing the taxonomic composition at genus level. The top20 genera are shown, representing 97.62% of all genera present. Genera are listed from the less abundant to the more abundant within each phylum (top to bottom), and ordered in ascending order within each phylum (Tenericutes, Fusobacteria, Bacteroidetes, Actinobacteria, and Firmicutes). Taxa barplots were colored according to each phylum (Tenericutes: gray-black, Fusobacteria: green, Bacteroidetes: purple, Actinobacteria: orange-yellow, and Firmicutes: blues). Furthermore, samples were ordered by the relative abundance of *Lactobacillus* in each woman in each group.

Women with HPV16 infection (*) and cervical cytology abnormalities are shown.

Abbreviations: LSIL: low-grade squamous intraepithelial lesions, HIV: human immunodeficiency virus, HPV: human papillomavirus, HPVN: HPV negative, HPVP: HPV negative, HSIL: high-grade squamous intraepithelial lesions, SNW: seronegative women, WLWH: women living with HIV
