# Supplementary material for: The vaginal microbiota of women living with HIV on suppressive antiretroviral therapy and its relation to high-risk human papillomavirus infection
Source: BMC Microbiol. 2023 Jan 19;23:21. doi: 10.1186/s12866-023-02769-1 (PMC9850673; doi:10.1186/s12866-023-02769-1)
Supplement: Supplementary file 12 — Additional file 12. The vaginal microbiota of women living with HIV and seronegative women with or without HR-HPV infection at species level (top 20 species). [file 12866_2023_2769_MOESM12_ESM.docx]

**Additional file 12: The vaginal microbiota of women living with HIV and seronegative women with or without HR-HPV infection at species level (top 20 species)**


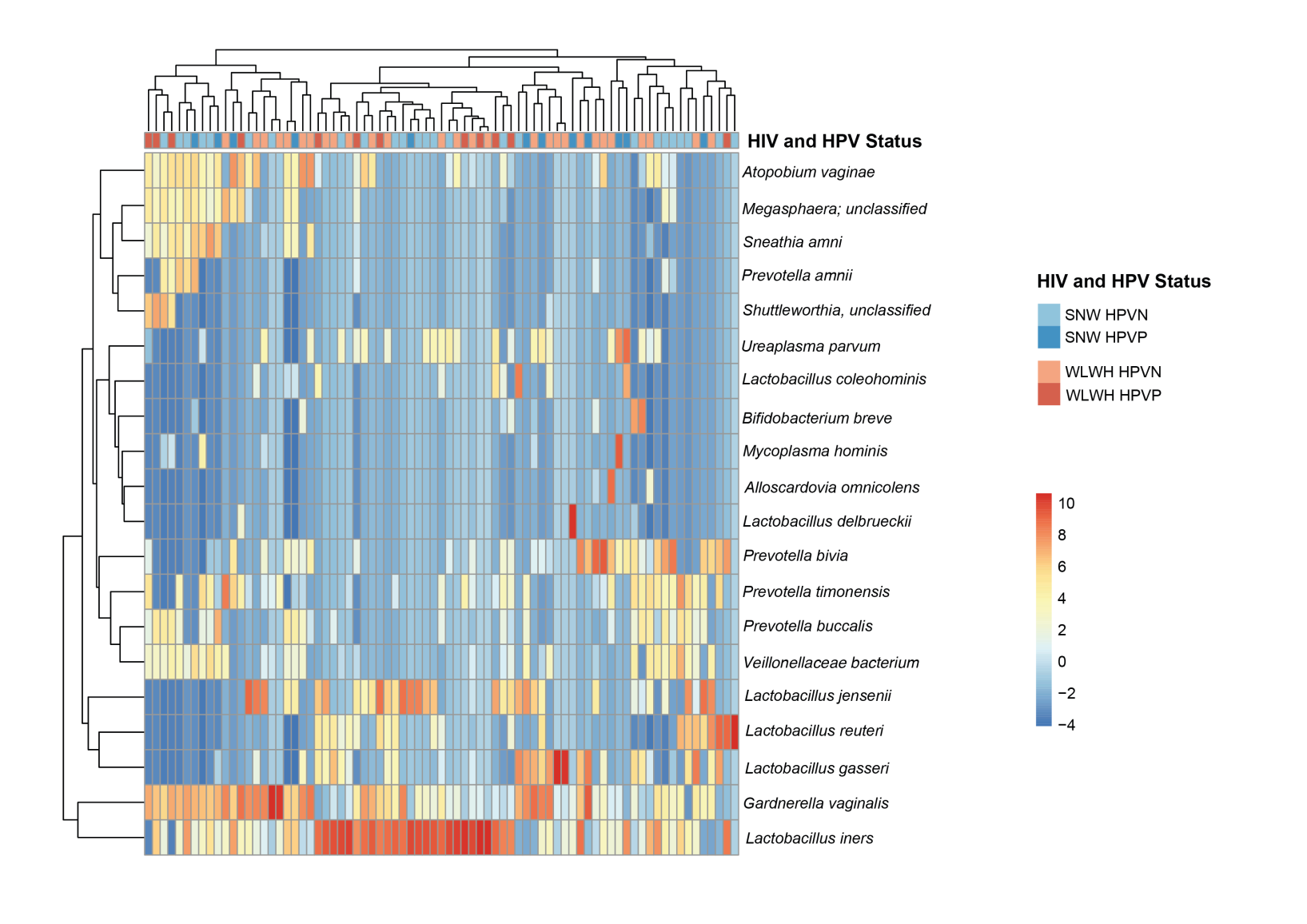


Legend:

Heatmap showing the top 20 species stratified by HIV and HPV status. The heatmap was generated using ampvis2 in R. Relative abundances were centered log-ratio transformed.

Abbreviations: HIV: human immunodeficiency virus, HPV: human papillomavirus, HPVN: HPV negative, HPVP: HPV negative, SNW: seronegative women, WLWH: women living with HIV
