# Supplementary material for: The vaginal microbiota of women living with HIV on suppressive antiretroviral therapy and its relation to high-risk human papillomavirus infection
Source: BMC Microbiol. 2023 Jan 19;23:21. doi: 10.1186/s12866-023-02769-1 (PMC9850673; doi:10.1186/s12866-023-02769-1)
Supplement: Supplementary file 13 — Additional file 13. Inverse correlations were found between Lactobacillus and Gardnerella, Prevotella, Dialister, and Atopobium. [file 12866_2023_2769_MOESM13_ESM.docx]

**Additional file 13: Inverse correlations were found between *Lactobacillus* and *Gardnerella*, *Prevotella*, *Dialister,* and *Atopobium***


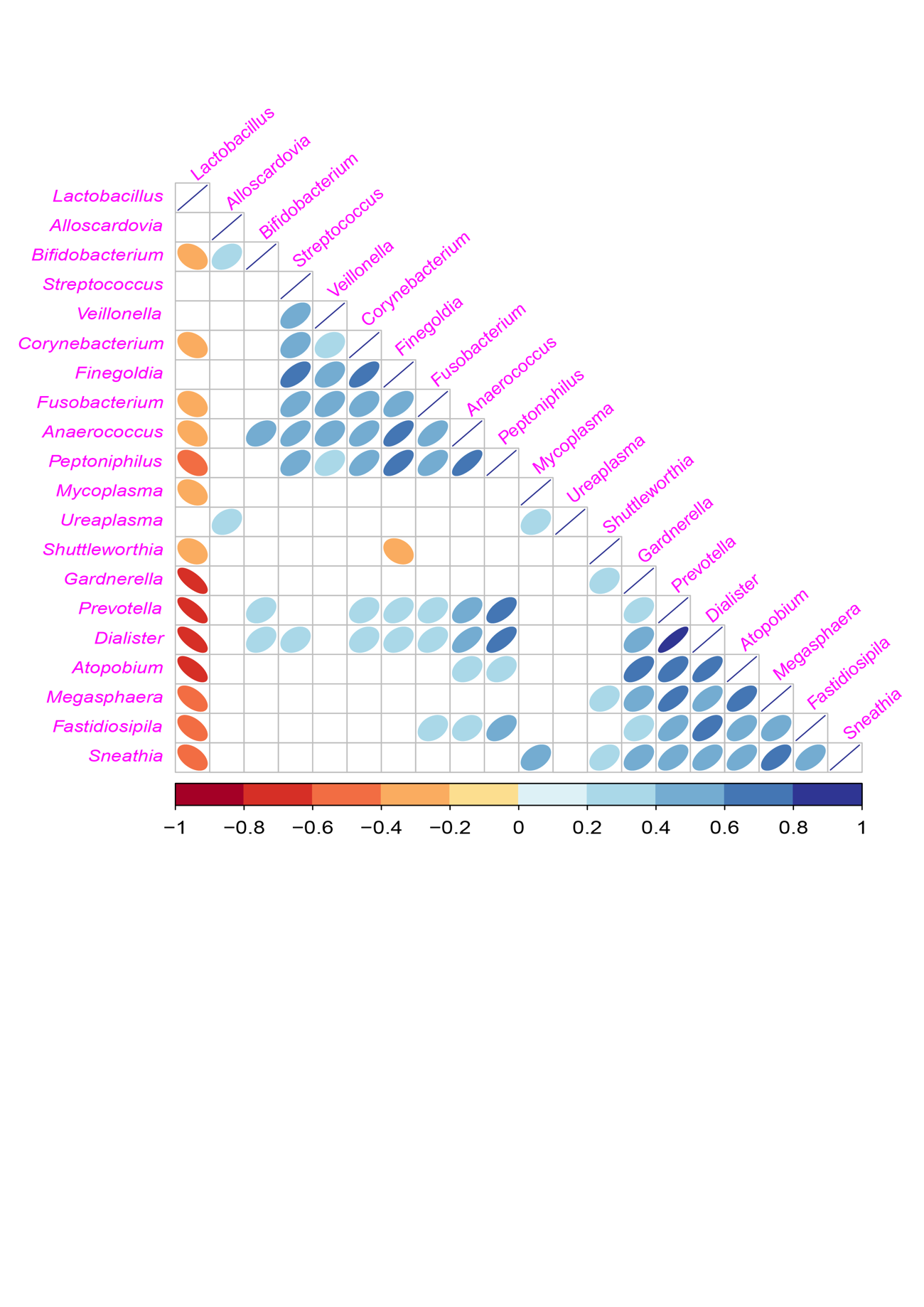


Legend:

Correlations between the top 20 genera were computed using Spearman test, and visualized using corrplot. Positive rho values are shown in blue, negative rho values shown in red, the intensity of the color is proportional to the strength of the rho value. Only *p* values< 0.01 are shown.
