# Supplementary material for: The vaginal microbiota of women living with HIV on suppressive antiretroviral therapy and its relation to high-risk human papillomavirus infection
Source: BMC Microbiol. 2023 Jan 19;23:21. doi: 10.1186/s12866-023-02769-1 (PMC9850673; doi:10.1186/s12866-023-02769-1)
Supplement: Supplementary file 14 — Additional file 14. Representative gating strategy. [file 12866_2023_2769_MOESM14_ESM.docx]

**Additional file 14: Representative gating strategy**


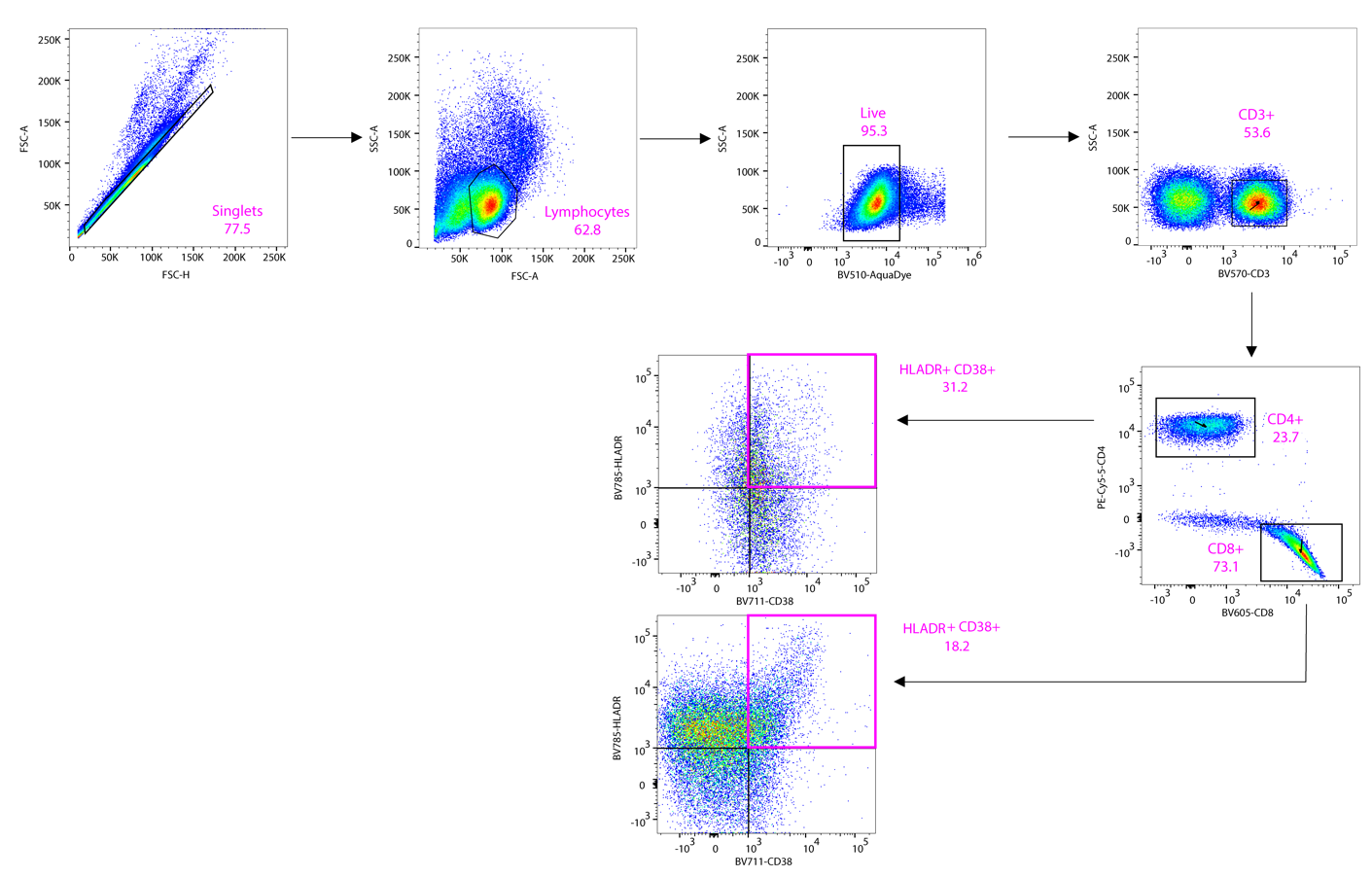


Legend:

This gating strategy was used for all samples. First fcs files were quality controlled using FlowAI, events were then gated for singlets (forward scatter area versus height) and morphology (forward versus side scatter). Next, dead cells were excluded (aqua dye negative events). Live cells were then gated on CD3+ and then on CD4+ or CD8+ T-cells. Immune activation was defined by the co-expression of CD38 and HLADR on total CD4+ and CD8+ T cells.
